# A review of flavonoids at the crossroads of plant defense: integrating biotic and abiotic stress tolerance through AI- and CRISPR/Cas-guided metabolic reprogramming

**DOI:** 10.3389/fpls.2026.1865665

**Published:** 2026-07-31

**Authors:** Aiman Hina, Asim Abbasi, Amna Chaudhry, Tayyaba Sanaullah, Muhammad Arshad, Hafiza Mehwish Sarwar, Sara Sarfaraz, Benjamin Karikari, Nyasha J. Kavhiza

**Affiliations:** 1 Ministry of Agriculture (MOA) National Centre for Soybean Improvement, State Key Laboratory for Crop Genetics and Germplasm Enhancement, Nanjing Agricultural University, Nanjing, China; 2 Seed Insect Management Laboratory, National Seed Research and Training Center, University of Agriculture, Faisalabad, Pakistan; 3 Department of Food Engineering and Biotechnology, Faculty of Agricultural, Environmental and Food Sciences, Free University of Bozen-Bolzano, Bozen-Bolzano, Italy; 4 Department of Botany, Government Sadiq College Women University, Bahawalpur, Pakistan; 5 Department of Entomology, University of Agriculture, Faisalabad, Pakistan; 6 Department of Bioinformatics, Fatima Jinnah Women University, Rawalpindi, Pakistan; 7 Department of Agronomy and Horticulture, Institute of Agriculture and Natural Resources, University of Nebraska-Lincoln, St, Lincoln, NE, United States; 8 Department of Environmental Management, Institute of Environmental Engineering, RUDN University, Moscow, Russia

**Keywords:** artificial intelligence, combined stresses, flavonoid regulatory networks, genome editing, phenylpropanoid metabolism, predictive metabolic engineering, stress adaptation

## Abstract

Flavonoids are multifunctional phenylpropanoid-derived metabolites that occupy a central position in plant adaptation to environmental stress. Beyond their established roles in antioxidant protection, they contribute to defense against pathogens and herbivores, signaling processes, and physiological acclimation to adverse environmental conditions. Although flavonoid responses to individual biotic or abiotic stresses have been extensively investigated, considerably less attention has been given to how flavonoid-associated regulatory networks function when multiple stresses occur simultaneously. This gap is particularly important because crops in agricultural systems are routinely exposed to overlapping biotic and abiotic challenges that generate distinct physiological, transcriptional, and metabolic responses. This review synthesizes current knowledge of flavonoid biosynthesis, structure-activity relationships, and the regulatory mechanisms governing flavonoid accumulation under diverse stress conditions. Particular emphasis is placed on the reorganization of flavonoid-associated networks under combined stress, including signaling crosstalk, pathway competition, metabolic trade-offs, and flux allocation that collectively shape adaptive responses. This review further evaluates how artificial intelligence can support identification of regulatory targets and pathway bottlenecks, how integration with CRISPR/Cas technologies may facilitate more precise manipulation of flavonoid biosynthesis, and how iterative Design–Build–Test–Learn (DBTL) frameworks could improve predictive flavonoid engineering through continuous integration of computational prediction and experimental validation. By integrating advances in stress biology, computational prediction, genome engineering, and iterative DBTL frameworks, this review outlines a roadmap for predictive reprogramming of flavonoid networks under combined stress and the development of crops with improved resilience to increasingly complex environmental conditions.

## 1 Introduction

Flavonoids are a structurally diverse group of phenylpropanoid-derived specialized metabolites that are widely distributed throughout plants (Khalid et al., 2019; Roy et al., 2022). More than 8,000 flavonoid compounds have been identified, including flavonols, flavones, flavanones, anthocyanins, and isoflavones (Xiao and Kai, 2012). Structural diversification through hydroxylation, methylation, prenylation, and glycosylation generates extensive chemical and functional variability among flavonoid subclasses (Crozier et al., 2006). Although traditionally associated with pigmentation, reproduction, and development (Griesbach, 2005; Diwan and Panche, 2016), flavonoids are now recognized as central regulators of plant responses to environmental stress.

Environmental constraints such as drought, salinity, temperature extremes, and ultraviolet radiation frequently stimulate flavonoid biosynthesis, where these metabolites contribute to antioxidant defense, membrane stabilization, and signaling regulation (Manghwar and Zaman, 2024; Nadeem et al., 2025; Mustafa et al., 2026). Flavonoids also participate in defense against pathogens and herbivores through antimicrobial activity, allelopathic interactions, feeding deterrence, and modulation of inducible defense responses (Samanta et al., 2011; Cesco et al., 2012). Their integration with redox homeostasis, phytohormone signaling, and environmental sensing pathways positions flavonoid metabolism at the interface between stress perception and adaptive responses (Mathesius, 2018).

Despite extensive investigation of flavonoids-mediated stress responses, much of the current knowledge has been derived from studies examining individual stress factors. This reductionist framework fails to reflect field conditions, where crops are commonly exposed to multiple biotic and abiotic stresses simultaneously (Pandey et al., 2017; Preece et al., 2024). Comparative analyses of demonstrate that combined stresses generate unique physiological, transcriptional, and metabolic states rather than simple additive effects (Suzuki et al., 2014; Ramegowda and Senthil-Kumar, 2015). For example, flavonoid profiles observed under combined heat-salinity stress differ substantially from those induced by salinity alone, indicating that stress combinations reshape phenylpropanoid flux and metabolic prioritization (Liu et al., 2013; Martinez et al., 2016). Likewise, transcriptomic studies have shown that many multifactorial stress-responsive genes are absent from corresponding single-stress datasets, highlighting extensive regulatory reprogramming under combined environmental conditions (Nawaz et al., 2023). These findings indicate that flavonoid-mediated stress adaptation is an emergent property of interconnected signaling, regulatory, and metabolic networks rather than the consequence of isolated gene or metabolite activities.

The increasing availability of transcriptomic, metabolomic, proteomic, genomic, and epigenomic datasets provides unprecedented opportunities to reconstruct regulatory interactions, identify network bottlenecks, and characterize the dynamic processes governing flavonoid accumulation and function (Helmy et al., 2020; Khan et al., 2026; Murtaza et al., 2026). However, converting these large and heterogenous datasets into mechanistic understanding and practical breeding strategies remains a major challenge. Accordingly, this review is guided by four key questions: (i) How does the structural diversity of flavonoids shape their biological functions in plant defense? (ii) How are flavonoid regulatory and metabolic networks organized across biotic and abiotic stress, and how do these networks converge and reprogram under combined stress conditions? (iii) How can artificial intelligence (AI), supported by multi-omics data, decipher flavonoid regulatory networks and prioritize engineering targets? (iv) How can AI-guided CRISPR/Cas (Clustered Regularly Interspaced Short Palindromic Repeats–CRISPR-associated proteins) technologies enable predictive reprogramming of flavonoid regulatory networks to enhance crop resilience?

Recent advances in artificial intelligence and CRISPR/Cas genome editing offer complementary solutions to these challenges. AI can integrate multi-dimensional biological datasets to infer regulatory relationships, predict metabolic bottlenecks, guide efficacy prediction (Kim et al., 2020; Xiang et al., 2021), identify guides tailored to these novel systems (Xiao et al., 2021), simulate the cellular repair processes that follow genome editing- induced cuts (Thomson et al., 2026), and prioritize intervention points that are difficult to resolve through conventional analyses (Bai et al., 2024). In parallel, CRISPR/Cas technologies enable precise manipulation of genes controlling flavonoid biosynthesis, regulation, transport, and metabolic flux (Zhu et al., 2020; Asmamaw and Zawdie, 2021; Gao, 2021; Nawaz et al., 2025). Together, these complementary approaches create opportunities to move beyond descriptive studies toward predictive reprogramming of flavonoid regulatory networks under complex environmental conditions.

While previous reviews have summarized flavonoid biosynthesis, transcriptional regulation, or metabolic-engineering as separate topic (Sheng et al., 2020; Mao et al., 2025), this review adopts an integrative perspective that connects these areas within the broader context of plant adaptation to complex environmental stresses. By bringing together evidence from stress biology, network regulation, artificial intelligence, and genome editing, this review highlights a conceptual framework for understanding and engineering flavonoid-mediated stress resilience.

To structure this synthesis, Section 2 summarizes the flavonoid biosynthetic pathways and its major regulatory checkpoints. Section 3 examines structure-activity relationships (SAR) underlying flavonoid-mediated defense against biotic stress, whereas Section 4 discusses the regulatory mechanisms through which flavonoids contribute to abiotic stress adaptation. Section 5 synthesizes current knowledge on the systems-level reprogramming of flavonoid networks under combined stress conditions. Section 6 highlights how artificial intelligence, supported by multi-omics integration and network biology, facilitates the discovery of regulatory hubs and candidate engineering targets. Section 7 discusses AI-guided CRISPR/Cas strategies and present a Design–Build–Test–Learn (DBTL) framework for predictive metabolic reprogramming of flavonoid networks. Finally, Section 8 examines the translational potential, current challenges, and future prospects for implementing AI- and CRISPR/Cas-enabled flavonoid engineering in crop improvement programs. As advances in AI, multi-omics, and genome-editing continue to converge, integrating these technologies with flavonoid biology will be instrumental in developing resilient crops capable of adapting to increasingly unpredictable environmental conditions.

## 2 Literature search strategy

Recent literature of this narrative review was identified through searches of Web of Science Core Collection, PubMed, Science Direct, and Google Scholar using combinations of keywords related to flavonoid biosynthesis, phenylpropanoid pathway, structure-activity relationships, biotic stress, abiotic stress, combined stress, secondary metabolism, redox homeostasis, phytohormone signaling, metabolic flux, metabolic branch points, pathway competition, metabolic trade-off, multi-omics, artificial intelligence, machine learning, CRISPR/Cas systems, and metabolic engineering. The search primarily considered studies published up to 2026 and was further supplemented through backward and forward citation tracking of key articles and recent reviews. Studies were selected based on their relevance to flavonoid-mediated stress adaptation, regulatory networks, and emerging engineering strategies in plants. Priority was given to peer-reviewed articles reporting experimental evidence, mechanistic insights, omics-based analyses, functional characterization of biosynthetic and regulatory genes, and advances in AI-assisted target identification and genome engineering approaches. The selected literature was critically evaluated and integrated to provide a conceptual framework linking flavonoid biology with next-generation strategies for improving crop resilience under complex environmental conditions.

## 3 Flavonoid biosynthesis pathways in plants

**Figure 1:**
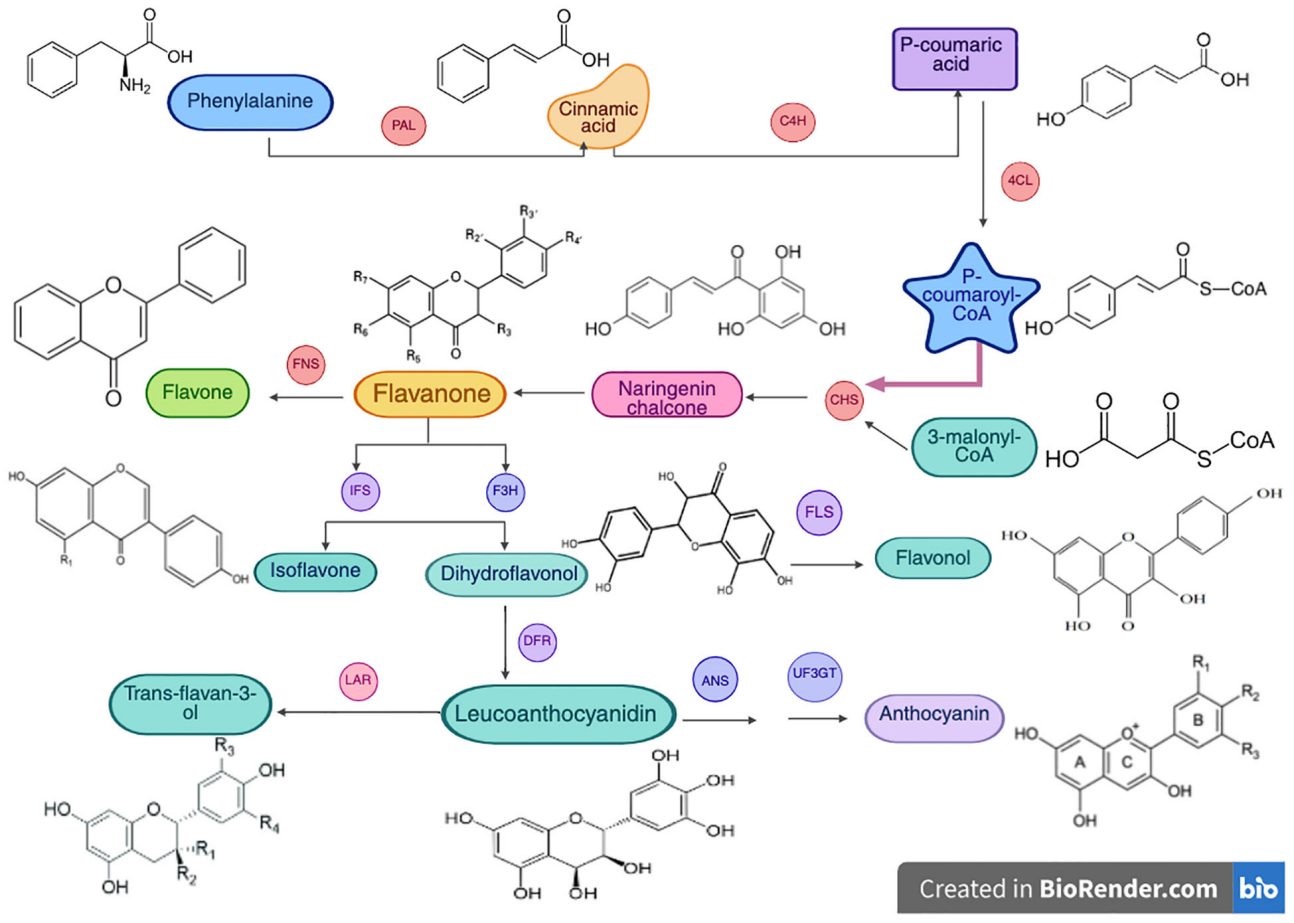
Schematic overview of the flavonoid biosynthetic pathway in plants. Flavonoid biosynthesis is initiated through the phenylpropanoid pathway, where phenylalanine is deaminated by phenylalanine ammonia-lyase (PAL) to form cinnamic acid. Cinnamic acid is subsequently hydroxylated by cinnamate 4-hydroxylase (C4H) to generate p-coumaric acid, which is then activated by 4-coumarate-CoA ligase (4CL) to produce p-coumaroyl-CoA, the central precursor for flavonoid biosynthesis. The pathway proceeds through the coordinated activities of key enzymes, including chalcone synthase (CHS), chalcone isomerase (CHI), isoflavone synthase (IFS), flavone synthase (FNS), flavanone 3-hydroxylase (F3H), flavonol synthase (FLS), dihydroflavonol 4-reductase (DFR), anthocyanidin synthase (ANS), leucoanthocyanidin reductase (LAR), and UDP-glucose:flavonoid 3-O-glucosyltransferase (UF3GT). Sequential enzymatic modifications generate a diverse array of flavonoid subclasses, including flavones, flavonols, isoflavonoids, anthocyanins, and proanthocyanidins. These metabolites contribute to numerous biological functions, including plant growth and development, stress adaptation, pigmentation, signaling, and defense against biotic and abiotic challenges.

Flavonoids are synthesized through the phenylpropanoid pathway, a highly conserved metabolic network that is spatially and temporally regulated across specialized tissues and cell types (Mathesius, 2018). The pathway is initiated by the deamination of phenylalanine-by-phenylalanine ammonia-lyase (PAL), followed by sequential hydroxylation and activation by cinnamate-4-hydroxylase (C4H) and 4-coumarate-CoA ligase (4CL) to generate p-coumaroyl-CoA (**Figure 1**). This intermediate represents the principal entry point into flavonoid biosynthesis, where chalcone synthase (CHS), the first committed and rate-limiting enzyme of the pathway, catalyzes the condensation of one molecule of p-coumaroyl-CoA with three molecules of malonyl-CoA to produce naringenin chalcone (Deng et al., 2018). Chalcone isomerase (CHI) subsequently converts naringenin chalcone into naringenin, a central metabolic hub from which virtually all major flavonoid subclasses are derived (Tohge et al., 2017). Thus, although the upstream reactions are relatively conserved, the biological diversity of flavonoids largely arises from downstream branch-point reactions that generate structurally distinct metabolites with specialized physiological functions.

Following naringenin formation, the pathway diverges into multiple competing branches through the coordinated activity of several downstream enzymes. Flavanone 3 β-hydroxylase (F3H) converts flavanones into dihydroflavonol, which serve as common intermediates for the biosynthesis of flavonols, anthocyanins and proanthocyanidins. Dihydroflavonol 4-reductase (DFR) channels metabolic flux towards leucoanthocyanidin, which are subsequently oxidized by anthocyanidin synthase (ANS) to produce anthocyanidins responsible for red, purple, and blue coloration in flowers, fruits, and vegetative tissues (Teles et al., 2018). Alternatively, leucoanthocyanidin reductase (LAR) catalyzes the formation of flavan-3-ols that contribute to antioxidant capacity, defence against herbivores and pathogens, and protection against environmental stresses (Rana and Gulliya, 2019). Importantly, these downstream reactions should not be viewed as independent linear pathways but as interconnected metabolic branches that compete for common substrates. The partitioning of carbon among flavonols, anthocyanins and proanthocyanidins is dynamically adjusted in response to developmental cues and environmental signals. Consequently, fluctuations in the activity of branch-point enzymes such as DFR, LAR and other competing enzymes can substantially alter flavonoid composition, influencing whether plants preferentially accumulate pigments, antioxidants or defence-related metabolites. These metabolic plasticity enables plants to optimize resource allocation according to specific physiological demands but also introduces considerable regulatory complexity into flavonoid biosynthesis.

The regulation of flavonoid biosynthesis extends beyond enzyme activity alone. CHS serves as a major control point governing carbon entry into the flavonoid pathway, whereas subsequent metabolic flux is coordinated through multilayered interactions among enzyme abundance, substrate availability, developmental programs and environmental signals (Trantas et al., 2015; Diwan and Panche, 2016). Studies shows that transcriptional regulators, particularly MYB, bHLH and WD40 proteins, integrate these developmental and stress-associated signals to coordinate pathway activity and maintain metabolic homeostasis (Yonekura-Sakakibara et al., 2019). Such hierarchical regulation enables rapid reconfiguration of flavonoid biosynthesis during biotic and abiotic stress while minimizing unnecessary metabolic expenditure. This regulation hierarchy highlight that flavonoid production reflects dynamic carbon allocation rather than simple constitutive metabolite accumulation.

Despite substantial advances in elucidating the flavonoid biosynthetic pathway, several technical and biological challenges continue to limit its effective manipulation. Enhancing flavonoid production is frequently constrained by competition among parallel metabolic branches, feedback regulation, limited precursor availability and incomplete understanding of flux control at key branch points. Moreover, the accumulation of structurally related flavonoids complicates metabolic identification, quantification and purification. While tissue-specific expression patterns and environmental variability often led to inconsistent metabolite profiles across species and experimental conditions. These limitations indicate that modifying individual biosynthetic enzymes rarely produces predictable changes in flavonoid composition because pathway behavior emerges from coordinated interactions among metabolic, transcriptional and cellular regulatory networks (Sajid et al., 2021). Collectively, current evidence demonstrates that flavonoid biosynthesis is governed by a highly dynamic and interconnected regulatory systems rather than a simple linear enzymatic pathway. Although the core biochemical reactions are well characterized, predicting flavonoid accumulation under complex environmental conditions remains challenging because metabolic flux is continuously reshaped by interactions among branch-point enzymes, regulatory networks and stress-responsive signaling pathways. Addressing these complexities will require systems-level approaches that integrate transcriptomic, metabolomic and regulatory information to identify the mechanisms controlling flavonoid reprogramming. Such knowledge provides the mechanistic foundation for the subsequent discussion of flavonoid-mediated stress adaptation, AI-assisted network analysis and precision metabolic engineering strategies aimed at improving crop resilience and the production of high-value bioactive compound (Davies et al., 2024).

## 4 Structure-guided functional diversification of flavonoids in biotic stress defense

Flavonoids share a conserved C6-C3-C6 backbone composed of two phenyl rings linked by a 3-carbonated heterocyclic ring (Boojar, 2019; Al Aboody and Mickymaray, 2020; Pei et al., 2020). Structural modifications to this core scaffold generate remarkable chemical diversity, giving rise to major flavonoids subclasses including flavanols, flavones, isoflavones, anthocyanidins, flavanones, and flavan-3-ols (Dudnik et al., 2018; Ciumărnean et al., 2020; Gujar and Wairkar, 2020; Kopustinskiene et al., 2020). These structural variations determine physiochemical properties such as polarity, stability, and molecular interactions, ultimately shaping the biological functions of individual flavonoids.

**Figure 2:**
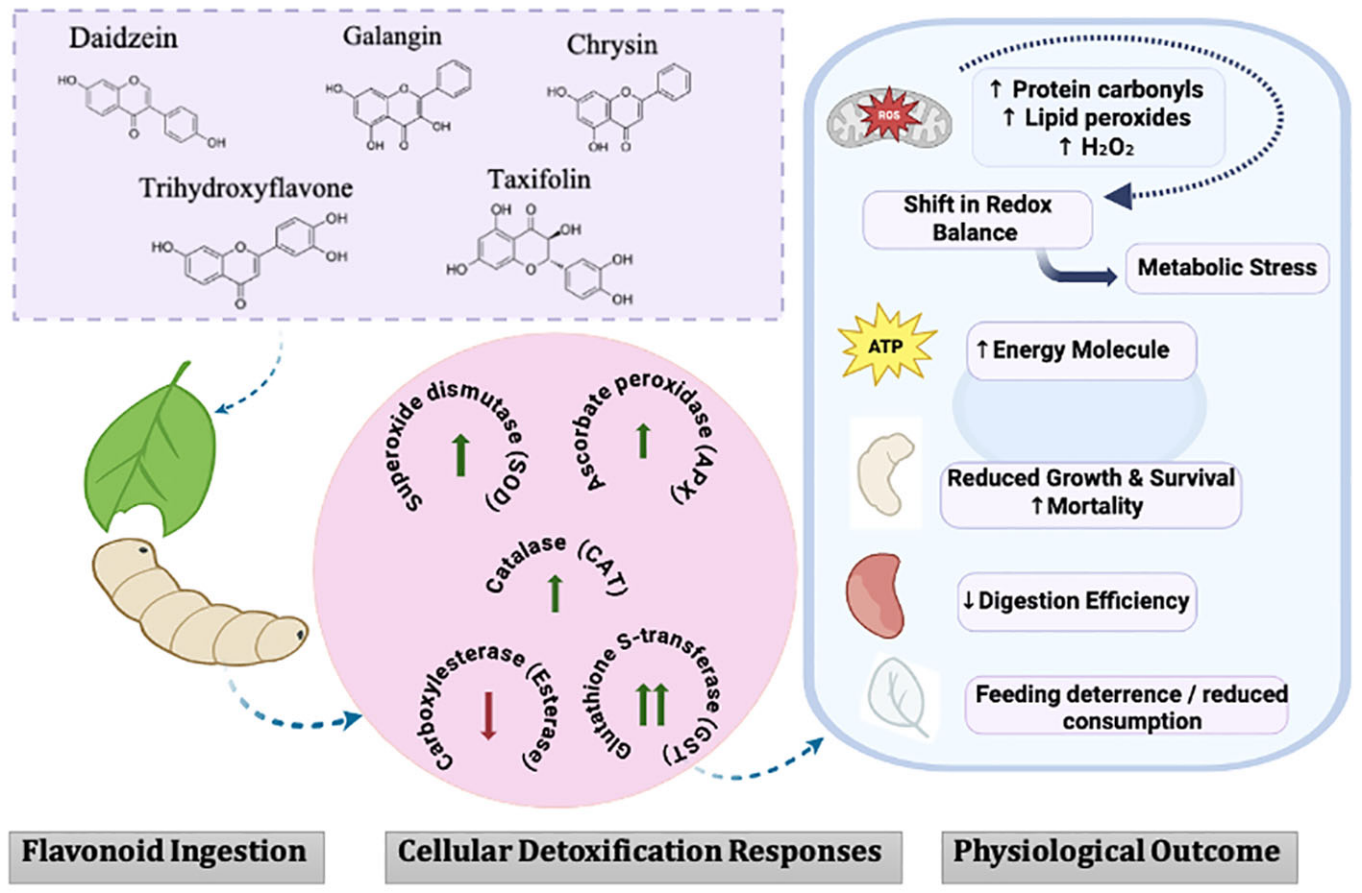
Dietary flavonoids ingested by insect larvae trigger cellular detoxification responses that modulate antioxidant enzyme activity, leading to oxidative stress and physiological disruption. Upon ingestion of flavonoid-rich plant material (e.g., daidzein, catechin, trihydroxyflavone, taxifolin, chrysin, galangin), midgut cells upregulate antioxidant enzymes (APX, SOD, CAT, GST) while downregulating esterases, resulting in reactive oxygen species (ROS) accumulation and elevated oxidative damage markers (protein carbonyls, lipid peroxides, H_2_O_2_). This redox imbalance diverts metabolic energy toward defense mechanisms, causing reduced larval growth and increased mortality. Arrows indicate the direction and magnitude of enzyme modulation: green upward arrows (↑) represent enzyme activation, red downward arrows (↓) denote enzyme inhibition, and double green arrows (↑↑) highlight strong or dominant induction, particularly for glutathione S-transferase (GST).

Rather than acting as broad-spectrum toxins, flavonoids function as structurally programmable defense metabolites. Their activity depends on scaffold architecture, hydroxylation pattern, glycosylation status, methoxylation, tissue localization, concentration, and the physiology of the target organism (Yuan et al., 2019; Zhao et al., 2021a; Franceschini Sarria et al., 2022; Puri et al., 2022). Consequently, relatively small structural modifications can markedly alter target specificity, potency, selectivity, persistence, and mode of action, establishing clear structure-activity relationship (SAR) that underpin flavonoid-mediated defense (Xie et al., 2015; Shamsudin et al., 2022) (**Figure 2**).

Plant encounter a wide range of biotic stresses, including microbial pathogens, insect herbivores, viruses, and plant-parasitic nematodes (Akram et al., 2025; Haq et al., 2026). Across these interactions, flavonoids rarely rely on a single defense mechanism. Instead, they generate complementary protective responses, including feeding deterrence, digestive inhibition, oxidative disruption, membrane destabilization, developmental toxicity, antimicrobial activity, and modulation of defense signaling pathways (**Table 1**). Comparative studies further demonstrate that plants selectively accumulate chemically optimized scaffolds rather than broadly activating secondary metabolism (**Table 2**). This selective deployment reflects an evolutionary refined defense strategy that balances metabolic cost with protective efficacy while enabling adaptation to diverse biotic challenges (Mierziak et al., 2014; Nabil-Adam et al., 2023). Developing predictive SAR frameworks that integrate structural chemistry with molecular and ecological responses remains a major challenge and represent an important opportunity for future flavonoid research.

**Table 1:** Structure–activity relationships (SAR) of major flavonoid subclasses.

Flavonoid subclass	Representative compounds	Key SAR feature(s)	Typical concentration range (μM)	Representative experimental systems	References
Flavonols	Quercetin, Kaempferol, Myricetin	C2=C3 double bond, 4-oxo group, 3-OH substitution; catechol B-ring enhances activity	1–50	Cell culture, oxidative stress, inflammation, metabolic studies	(Chen et al., 2018; Safe et al., 2021; Tsuji et al., 2013)
Flavones	Luteolin, Apigenin, Baicalein	C2=C3 double bond and 4-oxo group; B-ring hydroxylation influences activity	5–50	Cell culture and inflammation models	(Safe et al., 2021; Tsuji et al., 2013)
Flavanones	Naringenin, Hesperetin, Eriodictyol	Saturated C2–C3 bond; catechol B-ring improves activity	10–200	Cell culture and rodent studies	(Chen et al., 2018; Safe et al., 2021)
Flavan-3-ols	Catechin, Epicatechin, EGCG	Multiple hydroxyl groups; galloylation enhances activity	1–100	Oxidative stress and inflammation models	(Safe et al., 2021; Tsuji et al., 2013)
Anthocyanidins/Anthocyanins	Cyanidin, Delphinidin, Malvidin	Flavylium cation structure; hydroxylation and methoxylation patterns affect activity	5–200	Cell culture and metabolic studies	(Panche et al., 2016; Safe et al., 2021)
Isoflavones	Genistein, Daidzein, Glycitein	B-ring attached at C3 position; hydroxylation pattern influences receptor binding	1–100	Hormone-responsive and metabolic models	(Panche et al., 2016)
Chalcones	Isoliquiritigenin, Butein, Xanthohumol	α,β-Unsaturated carbonyl system	1–50	Cell culture and animal studies	(Chen et al., 2018; Safe et al., 2021)
Prenylated flavonoids	Sophoraflavanone G, Xanthohumol	Prenylation increases lipophilicity and uptake	0.5–25	Cell culture and inflammation models	(Chen et al., 2018; Tang et al., 2025)
Methoxylated flavonoids	Tangeretin, Nobiletin, Sinensetin	Methoxylation improves stability and permeability	5–100	Neuroprotective and metabolic models	(Chen et al., 2018; Tsuji et al., 2013)
Glycosylated flavonoids	Rutin, Hesperidin, Isoquercitrin	Glycosylation enhances solubility but may reduce intrinsic activity	10–500	Cell culture and gut metabolism studies	(Chen et al., 2018; Tang et al., 2025)

**Table 2:** Experimental evidence for flavonoid-mediated plant resistance and adaptation to biotic and abiotic stresses.

Flavonoid/Class	Target Organism/Stress Factor	Experimental System	Structural Form	Treatment/ Dose*	Major Biological Effect	Proposed Mechanism	Reference
Luteolin, Genistein	*Acyrthosiphon pisum*	Artificial diet bioassay	Aglycones	NR	Reduced feeding activity	Antifeedant activity affecting host acceptance	(Goławska and Łukasik, 2012)
Quercetin, Naringenin	*Acyrthosiphon pisum*	Feeding and life-history assays	Aglycones	Species-dependent	Reduced growth, fecundity and longevity	Interference with feeding behaviour and nutrient utilization	(Riddick, 2024)
Maackiain, Judaicin	*Helicoverpa armigera*	Larval feeding assay	Isoflavonoids	NR	Reduced larval weight gain	Growth inhibition and reduced food conversion efficiency	(Simmonds and Stevenson, 2001)
Pinocembrin, Quercetin	*Spodoptera frugiperda*	Diet incorporation assay	Aglycones	NR	Reduced larval survival and biomass	Feeding deterrence and toxicity	(Diaz Napal and Palacios, 2015)
Maysin	*Helicoverpa zea*	Natural maize resistance study	Flavone glycoside	Endogenous accumulation	Delayed caterpillar development	Reduced digestibility and nutrient acquisition	(Elliger et al., 1981)
3-Deoxyanthocyanidins	*Spodoptera frugiperda*	Feeding assay	Anthocyanidins	NR	Increased mortality and reduced growth	Gut disruption and oxidative stress	(Lesko, 2023)
Daidzein, Genistein	*Meloidogyne incognita*	Resistant vs susceptible cultivars	Isoflavones	Endogenous accumulation	Enhanced resistance response	Phytoalexin-mediated defense activation	(Carpentieri-Pípolo et al., 2005)
Glyceollin	*Heterodera glycines*, *M. incognita*	Soybean defense studies	Pterocarpan phytoalexin	Induced accumulation	Reduced nematode success	Activation of localized defense responses	(Edens et al., 1995)
Flavones, Flavonols	*Meloidogyne incognita*	*In vitro* nematode assays	Mixed flavonoids	NR	Larval mortality	Nematocidal activity	(Samina et al., 2020)
Quercetin, Vitexin	Tobacco mosaic virus	Plant inoculation study	Flavonol and flavone glycoside	NR	Reduced systemic viral accumulation	SA signaling activation and antiviral defense priming	(Krcatović et al., 2008)
Quercetin	Tobacco mosaic virus	Gene expression and infection assays	Aglycone	NR	Reduced viral replication	Downregulation of Hsp70-associated replication processes	(Wang et al., 2022b)
Naringenin	Pectobacterium brasiliense	Virulence assays	Flavanone	μM range	Reduced soft rot severity	Quorum sensing inhibition and suppression of virulence genes	(Pun et al., 2025)
Naringenin	Vibrio harveyi, Escherichia coli	QS reporter assays	Flavanone	μM range	Reduced biofilm formation and virulence	Interference with autoinducer signaling	(Vikram et al., 2010)
Kaempferol, Quercetin	*Xanthomonas*spp.	*In vitro* antibacterial assays	Aglycones	NR	Growth inhibition	Membrane disruption and quorum sensing interference	(Li et al., 2022)
Genistein	*Xanthomonas axonopodis*, *Sclerotinia sclerotiorum*	Plant-pathogen interaction studies	Isoflavone	NR	Reduced disease severity	Enhanced antioxidant defenses and suppression of virulence pathways	(Zhang et al., 2022)
Kaempferol, Quercetin	Transgenic plant studies	Flavonols	Endogenous increase via F3H overexpression	Gene overexpression	Improved stress tolerance	ROS scavenging, ion homeostasis, heat-shock protein induction	(Jan et al., 2021)
Flavanones, Flavones, Flavonols	Transgenic plants	Mixed flavonoids	Increased endogenous accumulation	Gene overexpression	Enhanced thermotolerance	Improved antioxidant defense capacity	(Wu et al., 2025)
Rutin	Physiological stress studies	Flavonol glycoside	Endogenous accumulation	NR	Improved salt tolerance	ROS detoxification and K^+^ retention	(Ismail et al., 2015)
Luteolin, Apigenin	Metabolomic and transcriptomic studies	Flavones	Endogenous accumulation	NR	Improved drought adaptation	Upregulation of flavonoid biosynthesis and antioxidant pathways	(Gharibi et al., 2019)
Myricetin, Astragalin	Integrated omics studies	Flavonol and glycoside	Endogenous accumulation	NR	Enhanced antioxidant protection	Activation of phenylpropanoid-flavonoid networks	(Li et al., 2025)
Quercetin, Kaempferol	Physiological studies	Flavonols	Endogenous accumulation	NR	Reduced oxidative damage	UV screening and ROS scavenging	(Laoué et al., 2022)
Flavonols, Flavones, Anthocyanins	Multiple species studies	Mixed classes	Endogenous accumulation	NR	Protection against photooxidative stress	Light absorption and antioxidant activity	(Ferreyra et al., 2021)

* Treatment dose: Include concentration, application method, and exposure duration where available in the original publications. “NR” indicates not reported.

### 4.1 Flavonoid scaffold diversity underpins defensive specialization

A consistent pattern across biotic stress systems is the selective recruitment of specific flavonoid subclasses rather than generalized accumulation of phenolic metabolites. Across Lepidoptera, aphid, and beetle systems, resistance is consistently associated with flavonols, flavones, isoflavonoids, anthocyanins, and proanthocyanidins, although the defensive outcomes differ substantially among subclasses (Mallikarjuna et al., 2004; Nuessly et al., 2007). Flavonol-rich profiles containing quercetin and rutin correlate with enhanced resistance in wild groundnut, whereas C-glycosyl flavone such as maysin confer resistance in maize by impairing larval physiology and feeding efficiency through disruption of nutrient assimilation and oxidative balance (Mallikarjuna et al., 2004; Nuessly et al., 2007). In contrast condensed tannins such as procyanidins exert pronounced effects on reproductive fitness, including substantial suppression of fecundity in *Aphis craccivora* (Kaur and Ahmed, 2021). Similar specialization occurs in sorghum, where flavan-4-ols and 3-deoxyanthocyanidins accumulate rapidly following herbivory and contribute to resistance against stem borers and chewing insects through oxidative and digestive disruption (Lo et al., 1999). These observations indicate that flavonoid subclasses perform specialized defensive functions rather than redundant roles. Structural diversification enables plants to direct defense towards specific physiological targets while balancing defense effectiveness with metabolic cost.

The repeated occurrence of flavonols such as quercetin and myricetin across fungal, bacterial, insect and nematode interactions suggests that plants preferentially exploit chemically versatile scaffolds with broader target compatibility rather than evolving entirely stress-specific metabolites (Ramaroson et al., 2022). This recurrent scaffold reuse reflects an evolutionary trade-off between defense prioritization and metabolic efficiency. Quercetin-rich tissues suppress aphid feeding, inhibit bacterial DNA gyrase activity, interfere with fungal membrane integrity, and repel root-knot nematodes, illustrating broad-spectrum scaffold reuse across phylogenetically distinct antagonists (Plaper et al., 2003). Similar trends are observed in phytoalexin-mediated immunity, where isoflavonoids such as pisatin, maackiain, medicarpin, glyceollin, and coumestrol are closely associated with resistance in pea, chickpea, soybean, and alfalfa (Naoumkina et al., 2010). Notably, several of these scaffolds additionally contribute to insect deterrence and nematode resistance (Kour et al., 2024), supporting the concept of scaffold reuse across distinct biotic stress environments. Distinct flavonoid subclasses also show preferential deployment against specific biological targets. Flavonols and flavones frequently dominate insect antifeedant and digestive inhibitory responses, whereas isoflavonoids are more strongly associated with inducible antimicrobial defense (François et al., 2022). In nematode interactions, active metabolites include flavonols such as isorhamnetin, myricetin, fisetin, galangin, morin, as well as methoxylated flavones derived luteolin and apigenin (Samina et al., 2020).

Despite substantial progress, current evidence remains largely qualitative. Most studies have examined individual plant-pest or plant-pathogen interactions under controlled conditions. Consequently, it remains unclear whether these structure-activity relationships are conserved across species, environmental conditions, or combined biotic stresses. Future research should integrate comparative metabolomics, functional genetics, and quantitative structure-activity modelling. Such approaches will help establish predictive frameworks for flavonoid-mediated defense.

### 4.2 Structural modifications shape flavonoid bioactivity

#### 4.2.1 Hydroxylation

Among the major structural modifications, hydroxylation is one of the primary determinants of flavonoid bioactivity because it influences redox potential, hydrogen-bonding capacity, metal-chelation, and interactions with biological targets. Consequently, both the number and position of hydroxyl groups, rather than hydroxylation alone, govern flavonoid potency and target selectivity. Across phytophagous insects, hydroxylation at C5 and C7 of the A ring and at C3′ and C4′ of the B ring, together with the presence of a C4 carbonyl group, markedly enhances antifeedant activity (Ohmura et al., 2000; Morimoto et al., 2003). Additional hydroxyl substitution at C6 or C7 further strengthens deterrence, while asymmetric electron distribution and increased hydrogen-bonding potential likely facilitate interactions with insect gustatory receptors and digestive enzymes (Morimoto et al., 2003). These findings indicate that insect responses are determined not only by hydroxyl density but also by the spatial arrangement of hydroxyl groups. Thus, even subtle structural modifications can markedly alter flavonoid bioactivity and target specificity.

Hydroxylation also strongly influences enzyme inhibition and neurophysiological toxicity. In *Ostrinia furnacalis*, highly hydroxylated flavonols such as myricetin and quercetin together with baicalein inhibited chitinolytic enzymes more broadly than kaempferol, galangin, or chrysin, demonstrating scaffold-dependent activity rather than class-wide activity (Li et al., 2021). Similar SAR patterns occur in neurophysiological regulation. In *Spodoptera litura*, flavonoids containing multiple phenolic hydroxyl groups on the A and B rings exhibited stronger acetylcholinesterase inhibitory activity, with quercetagetin-7-O (6-O-caffeoyl-β-D-glucopyranoside) showing the highest inhibition potency (Li et al., 2020). Hydroxyl-rich flavonoids additionally interfere with digestive proteases and amylases, thereby reducing nutrient assimilation and larval growth (Chamani et al., 2025). In microbial systems, hydroxylation enhances metal-chelating capacity and membrane interaction, contributing to inhibition of fungal growth and bacterial respiration (Skadhauge et al., 1997). Collectively, these findings indicate that hydroxylation patterns directly influence flavonoid interactions with digestive, neural, oxidative, and developmental targets. However, the relationship between hydroxylation and bioactivity is not universally linear. Similar hydroxylation patterns can produce different biological outcomes depending on the flavonoid scaffold and the interacting organism. This variability suggests that hydroxylation interacts with other structural features to determine flavonoid function (Heim et al., 2002).

**Figure 3:**
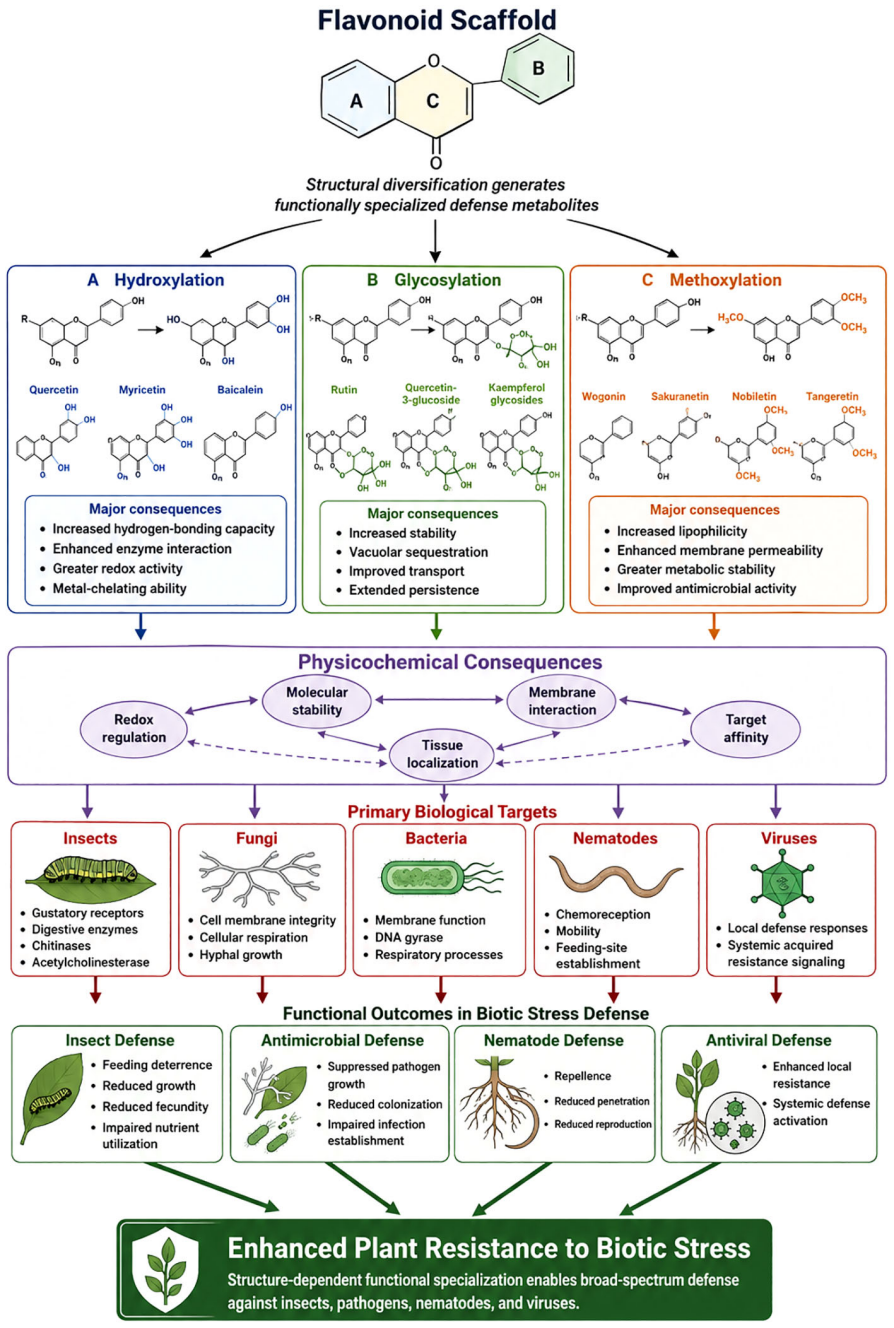
Structure–activity relationship (SAR) framework illustrating how flavonoid structural diversification through hydroxylation, glycosylation, and methoxylation modifies physicochemical properties, including redox activity, stability, localization, membrane interactions, and target affinity. These structural changes influence interactions with insects, fungi, bacteria, nematodes, and antiviral defense pathways, resulting in feeding deterrence, pathogen suppression, reduced nematode infection, and enhanced antiviral responses.

Hydroxylation also contributes to flavonoid-mediated oxidative disruption in insects. Although flavonoids function predominantly as antioxidants in plant tissues, ingestion within alkaline insect midguts can promote pro-oxidant activity through semiquinone formation and redox cycling (Barbehenn et al., 2005). Highly hydroxylated flavonoids such as daidzein, catechin, trihydroxyflavone, taxifolin, chrysin, galangin induce reactive oxygen species accumulation, protein carbonyls, lipid peroxides, H_2_O_2_ lipid peroxidation, and protein oxidation despite activation of insect antioxidant enzymes including ascorbate peroxidase (APX), glutathione S-transferase (GST), superoxide dismutase (SOD), and catalase (CAT) (Barbehenn et al., 2010). Rather than causing immediate lethality, sustained oxidative imbalance diverts metabolic resources towards detoxification and cellular repair. This reduces insect growth, development, and reproductive fitness. As illustrated in **Figure 3**, flavonoid-induction modulation of antioxidant and detoxification enzymes disrupts redox homeostasis, resulting in oxidative damage that ultimately reduces insect survival and fitness.

#### 4.2.2 Glycosylation

Glycosylation further refines flavonoid bioactivity by regulating chemical stability, transport, tissue localization, metabolic persistence and bioavailability (Yang et al., 2018). In contrast to aglycones, glycosylated flavonoids often exhibit improved vacuolar sequestration, enhanced phloem mobility, and reduced susceptibility to oxidative degradation. In resistant rice cultivars, glycoflavones preferentially accumulate in phloem sap, illustrating how tissue-specific allocation maximizes defensive efficiency while minimizing unnecessary metabolic costs (Grayer et al., 1993). Similarly, rutin, quercetin-3-glucoside, and kaempferol glycosides accumulate in epidermal tissues and vascular systems where they interfere with aphid probing and sustained feeding (Goławska and Łukasik, 2012). These observations suggest that glycosylation does more than stabilize flavonoids. It also directs their spatial distribution within plant tissues. This targeted allocation enables plants to concentrate defensive metabolites at sites most vulnerable to herbivore attack.

Furthermore, studies across insect systems suggest that glycosylated flavonols function as metabolically persistent antinutritional compounds rather than acute toxins. Flavonoid-rich soybean extracts suppress the growth and survival of *Trichoplusia ni* larvae. Comparable inhibitory effects have also been with quercetin 3-glucoside-supplemented diets (Harborne and Williams, 2000; Padial et al., 2023). These findings indicate that glycosylation prolongs the defensive function of flavonol beyond storage. By enhancing chemical stability, it enables sustained biological activity within the insect gut. Similar inhibitory effects observed in *Lymantria dispar* larvae exposed to flavonol-glycoside-rich pine needle extracts further suggest that glycosylated derivatives resist rapid detoxification while continuously interfering with digestion across taxonomically distinct insect systems (Beninger and Abou-Zaid, 1997).

Plants also exploit glycosylation as a reversible defense-storage mechanism. Flavonoids are frequently accumulated as inactive glycoside that become hydrolyzed into reactive aglycones following tissue disruption or herbivore ingestion (Morant et al., 2008; Pereira et al., 2024). This strategy allows plants to minimize autotoxicity while rapidly activating chemically reactive metabolites at sites of attack. Consequently, glycosylation provides both metabolic flexibility and rapid defence activation, allowing plants to balance protection with the avoidance of self-toxicity. Recent evidence further demonstrates that Uridine Diphosphate-dependent (UDP)-glycotransferases (UGTs) regulate resistance by controlling the accumulation of specific flavonoid glycosides rather than total flavonoid abundance, emphasizing that metabolic composition is often more important than overall flavonoid content (Zhou et al., 2025).

Glycosylated flavonoids are also repeatedly associated with microbial resistance. O-glycosylated flavonoids frequently accumulate in resistant genotypes challenged with fungal and oomycete pathogens and participate directly in host-pathogen interactions (Kröner et al., 2012; Ramaroson et al., 2022). For example, barley resistant to *Gibberella zeae* accumulates higher levels of kaempferol and naringenin glycosides than susceptible lines (Kumaraswamy et al., 2011). Citrus infected with *Candidatus Liberibacter asiaticus* similarly exhibits elevated flavonoid glycoside and hydroxycinnamic acid derivatives associated with bacterial restriction (Margaria et al., 2014). Collectively, these findings demonstrate that glycosylation is a key regulator of flavonoid function rather than a simple structural modification. It influences where flavonoids accumulate, how long they persist, and when they become biologically active. These properties allow plants to coordinate defense responses against a broad range of biotic stresses.

#### 4.2.3 Methoxylation

Methoxylation further diversifies flavonoid function by altering flavonoid lipophilicity, membrane permeability, metabolic stability, and enzyme-binding properties (Liu et al., 2022a). SAR analyses demonstrated that C8-methoxyl substitution enhances chitinolytic enzyme inhibition, with wogonin exhibiting stronger activity than baicalein (Li et al., 2021). The increased hydrophobicity associated with methoxylation enhances membrane interactions. It may also facilitate accumulation within pathogen-associated cellular compartments (Kour et al., 2024). Methoxylated flavonoids frequently display enhanced persistence and antimicrobial activity. Methyl substitution reduces their susceptibility to oxidative degradation and enzymatic conjugation, thereby prolonging their biological activity. Citrus infected with *Phytophthora citrophthora* accumulates polymethoxylated flavones such as nobiletin and tangeretin (François et al., 2022). This accumulation suggests that methoxylated scaffolds contribute to pathogen-responsive defense. Similarly, sakuranetin, a methoxylated flavanone phytoalexin in rice, exhibits potent antifungal activity against *Magnaporthe oryzae* by disrupting fungal membrane integrity and inhibiting appressorium formation (Cho and Lee, 2015).

Methoxylated derivatives of luteolin and apigenin also exhibit nematicidal activity (Samina et al., 2020). These findings suggest that methoxylation expand flavonoid defensive specialization and target selectivity (Kour et al., 2024). In bacterial systems, polymethoxylated flavones exhibit stronger membrane-disruptive activity than corresponding hydroxylated analogues due to increased lipophilicity and improved penetration of bacterial envelopes (Donadio et al., 2021). Collectively, these studies demonstrate that methoxylation extends flavonoid functional diversity beyond structural modification alone. By enhancing stability, membrane affinity, and target selectivity, methoxylation enables plants to strengthen defense against diverse biotic challenges.

### 4.3 Dose- and species-dependent outcomes of flavonoid SARs

A defining characteristic of flavonoid-mediated defense is its strong dependence on concentration and biological context. Rather than acting as constitutive toxins, flavonoids function as tunable defense metabolites. Their biological effects vary with dosage, target organism, and the ecological interaction. In insect systems, sub-threshold flavonoid concentrations may enhance feeding or survival, whereas elevated accumulation suppresses growth, development, reproduction, and digestive efficiency (Simmonds, 2003). Quercetin and pinocembrin can act as phagostimulants at low concentrations but become strongly deterrent and antinutritional at higher doses (Diaz Napal et al., 2010). Similarly, rutin and quercetin exerted limited negative effects on *Anthonomus grandis* and may even increase adult biomass under certain dietary conditions (Beck and Reese, 1976).

Defensive responses are also highly species specific. Certain flavonoids act as oviposition stimulants in specific insect taxa (Haribal and Renwick, 1996; Riddick et al., 2018). This response demonstrates that receptor specificity and sensory ecology strongly influence flavonoid bioactivity. Some insects sequester flavonoid glycosides into their integument, haemolymph, or salivary glands, repurposing plant defense compounds for protection against predators or for mating-related signaling (Bowers et al., 1993; Simmonds, 2003). These findings illustrate that flavonoid function is shaped not only by plant metabolism but also by the adaptive responses of the interacting organism.

Comparable context dependency occurs in microbial interactions. Potato tubers enriched in rutin and nictotiflorin exhibit resistance to *Pectobacterium atrosepticum* but remain susceptible to *Phytophthora infestans* (Naoumkina et al., 2010). This contrast indicate that antimicrobial efficacy depends on pathogen trophic strategy and infection niche rather than flavonoid abundance alone. Similar context dependency is evident in plant- nematode interactions. Flavonols such as kaempferol, myricetin, and quercetin repel *Radopholus similis* and *M. incognita*, whereas flavones (e.g., luteolin) and isoflavones (e.g., genistein and daidzein) exhibit more selective deterrent activity (Wuyts et al., 2006). In contrast to the broader antifeedant effects commonly reported against insect, these responses suggest that nematode chemoreception relies on more precise chemical recognition (Rasmann et al., 2012). Consistent with this observation, localized accumulation of glyceollin and coumestrol in soybean and lima bean roots coincides with nematode penetration and feeding-site establishment (Chin et al., 2018; Shah and Smith, 2020). This spatial accumulation indicates that flavonoids function not only as deterrent metabolites but also as regulators of localized root defense.

Viral infections also reveal the spatial specialization of flavonoid-mediated defense. In tobacco mosaic virus (TMV)-infected tobacco, quercetin accumulates preferentially at infection sites, whereas kaempferol accumulates in distal tissues associated with systemic acquired resistance (Treutter, 2006; Mierziak et al., 2014; Hu et al., 2023). This spatial distribution suggests functional partitioning between local containment and systemic defense signaling. These observations indicate that flavonoids frequently amplify host-mediated defense signaling pathways rather than directly targeting viral particles. Overall, these findings demonstrate that flavonoid-mediated defense is inherently context dependent. Biological outcomes arise from the combined effects of scaffold architecture, structural modifications, dosage, metabolite localization, persistence, and the biology of interacting organism. Together, these factors establish a mechanistic SAR framework that underpins flavonoid functional specialization during plant biotic stress.

Overall, flavonoid defense depends on more than accumulation of individual metabolites. The biological function of a flavonoid is influenced by its chemical structure, structural modifications, concentration, tissue distribution, and the characteristics of the interacting organism. As a result, the same flavonoid can perform different defensive roles in different plant-enemy interactions. This variability limits the broader application of structure-activity relationship established in individual experimental systems. Another important limitation is that most studies have examined flavonoid responses under a single stress. Whether these relationships remain valid when plants ae exposed to combined biotic and abiotic stresses is still unclear, as metabolic priorities continually shift to balance defense, growth and stress adaptation. A better understanding of how structural modifications influence flavonoid function under these dynamic conditions will be essential for identifying reliable engineering targets and enabling rational metabolic engineering to improve crop resilience.

## 5 Flavonoids regulatory networks in abiotic stress adaptation

Abiotic stress such as drought, salinity, extreme temperatures, and ultraviolet (UV) radiation severely impair plant growth by disrupting cellular homeostasis and promoting excessive accumulation of reactive oxygen species (ROS) (Patil et al., 2024; Murtiyaningsih et al., 2026). To maintain cellular integrity under these conditions, plants activate coordinated defense responses involving antioxidant metabolism, hormonal signaling, and transcriptional reprogramming (Sachdev et al., 2021). Within these adaptive networks, flavonoids function as important regulators of redox balance, photoprotection, membrane stability, and stress-associated signaling rather than merely end-products of secondary metabolism (Agati et al., 2012; Brunetti et al., 2013; Sharma et al., 2019). Importantly, many structural features that govern flavonoid activity during biotic interactions also influence abiotic stress adaptation. Variation in hydroxylation, methoxylation, glycosylation, acylation, and polymerization alter antioxidant potential, membrane affinity, subcellular localization, and photoprotective capacity, thereby generating substantial functional diversity among flavonoid subclasses (Rice-Evans et al., 1996; Agati et al., 2020). Consequently, abiotic stress tolerance depends less on generalized flavonoid accumulation than on selective reprogramming of structurally specialized flavonoid networks optimized for distinct physiological and ecological functions.

### 5.1 ROS scavenging and redox regulation

Oxidative imbalance is one of the earliest cellular responses to abiotic stress (Sharma et al., 2012). Excessive ROS production under drought, salinity, heat, and UV stress damages proteins, membrane, and photosynthetic complexes while also serving as a signaling cue for metabolic reorganization (Sachdev et al., 2021). Flavonoid accumulation under these conditions reflects a controlled redistribution of reducing power toward localized redox buffering rather than a generalized increase in antioxidant metabolites (Demidchik, 2018). Stress induced upregulation of PAL, 4CL, F3H, and flavonol synthase (FLS) across multiple species indicates that flavonoid biosynthesis is tightly coupled to oxidative stress signaling (Ghasemi et al., 2023; Park et al., 2023; Chen et al., 2025). Drought-tolerant rice genotypes accumulate significantly higher flavonoid levels than sensitive cultivars (Quan et al., 2016), while enrichment of catechin and flavonols in *Camellia sinesis* and *Sesamum indicum* correlates with enhanced radical scavenging efficiency under drought stress (Cheruiyot et al., 2008; Kermani et al., 2019). These protective effects are strongly influenced by flavonoid structure. Dihydroxylated flavonoids exhibit greater electron-donating capacity and stabilize hydroxyl radicals more efficiently than monohydroxylated derivatives, whereas their metal-chelating properties suppress ROS propagation through the Fenton reaction (Shomali et al., 2022). These observations highlight that antioxidant efficiency is determined by flavonoid structure rather than metabolic abundance alone, indicating that oxidative stress signaling selectively favors compounds with the greatest functional advantage under a given stress condition.

Flavonoids also function in concert with enzymatic antioxidants (Agati et al., 2020). Under salinity stress, elevated flavonoid accumulation is commonly accompanied by increased SOD and CAT activities, suggesting coordinated regulation of enzymatic and non-enzymatic antioxidant system (Garcia-Caparros et al., 2021; Shomali et al., 2022). Heat and UV stress further demonstrate that oxidative protection is achieved through different flavonoid profiles rather than a common antioxidant response. In soybean, thermotolerant genotypes selectively accumulate daidzein, apigenin, daidzin, genistein, glycitein, kaempferol 3-O-beta-glucoside, genistin, and naringenin derivatives under elevated temperatures (Chebrolu et al., 2016). These contrasting flavonoid profiles indicate that plants optimize flavonoid composition according to the source and location of oxidative stress rather than increasing all flavonoid classes uniformly. Whereas, UV RESISTANCE LOCUS 8 (*UVR8*)-mediated activation of chalcone synthase (CHS) promotes epidermal accumulation of quercetin glycosides and luteolin derivatives that simultaneously absorb UV radiation and restrict ROS diffusion into internal tissues (Favory et al., 2009; Dao et al., 2011). These findings show that ROS functions not only as a damaging molecule but also as a regulatory signal linking environmental stress perception with flavonoid biosynthesis. By interacting with antioxidant enzymes, photoreceptor-mediated signaling pathways, and transcriptional regulation of flavonoid biosynthesis, flavonoids help maintain redox homeostasis across diverse stresses. Rather than functioning as isolated antioxidants, flavonoids operate within coordinated regulatory network that integrate ROS signaling with metabolic and transcriptional responses to stress.

### 5.2 Flavonoid biosynthetic regulation and metabolic stabilization

Abiotic stress disrupts metabolic homeostasis not only through excessive ROS accumulation but also by impairing enzyme activity, protein stability, and the coordination of metabolic pathways (Zhao et al., 2025). Beyond their antioxidant functions, flavonoids contribute to stress adaptation by preserving enzymatic activity, supporting photosynthetic performance, and maintaining metabolic homeostasis under adverse environmental conditions (Brunetti et al., 2013; Agati et al., 2020). The activation of flavonoid biosynthetic pathways is itself a component of stress-induced metabolic reprogramming, redirecting carbon flux toward metabolites that stabilize cellular function under adverse conditions. Several studies demonstrate that stress-induced regulation of flavonoid biosynthetic enzymes directly contribute to enhanced tolerance. Rather than increasing flavonoid production indiscriminately, stress signaling selectively activates key branch-point enzymes, allowing plants to adjust both the quantity and composition of flavonoids according to metabolic demand. In tobacco, upregulation of *NtCHS1* increases flavonoid accumulation and improves salinity tolerance (Chen et al., 2019). Similarly, elevated expression of *LpFLS1* and *LpCHI1*, and *GmCHI4* in ryegrass and soybean sustains flavonoid biosynthesis under ionic stress (Cao et al., 2024; Zhang et al., 2024a). Overexpression of *F3H* in rice enhanced quercetin and kaempferol biosynthesis under salinity stress (Jan et al., 2021), while *SIbHLH22* overexpression in tomato promotes flavonoid accumulation under drought and salinity stress, albeit with developmental trade-offs such as reduced plant height and smaller leaves (Waseem and Li, 2019). These developmental penalties illustrate that enhanced flavonoid biosynthesis is not universally beneficial, but instead reflects a balance between stress tolerance and growth. Likewise, the *AeCHS* gene from *Abelmosschus esculentus* stimulated flavonoid biosynthesis in transgenic *Arabidopsis* under osmotic and salt stress (Wang et al., 2018), and CHS overexpression improved high-light tolerance by enhancing anthocyanin accumulation and photoprotection (Zhang et al., 2018). In addition, *GAS1* overexpression contributed to metabolic flux redistribution and improved tolerance to drought, heat, and salinity stress (Dong et al., 2020). These genetic studies suggest that stress tolerance depends on coordinated regulation of multiple flavonoid biosynthetic steps rather than modification of a single enzyme. Beyond secondary metabolism, flavonoids help preserve core physiological processes associated with photosynthesis, nitrogen assimilation, and cellular energy balance, thereby linking specialized metabolism with primary metabolic function. Exogenous naringenin application in *Phaseolus vulgaris* alleviated salinity-induced physiological disruption by maintaining photosynthetic efficiency, nitrogen metabolism, and intracellular redox balance (Yildiztugay et al., 2020). These findings show that flavonoids support stress adaptation not simply through ROS detoxification but by maintaining metabolic coordination across multiple physiological processes. This broader regulatory role explains why manipulation of flavonoid biosynthetic genes often improves stress tolerance beyond changes in antioxidant capacity alone.

### 5.3 Membrane stabilization and lipid protection

Abiotic stress-induced oxidative and ionic disturbance compromise membrane integrity through lipid peroxidation and damage to membrane-associated proteins and transport systems (Van Zelm et al., 2020). Flavonoids mitigate these effects not only through antioxidant activity but also via direct interactions with lipid bilayers. Their amphipathic nature enables interactions with phospholipid headgroups and acyl chains, thereby influencing membrane fluidity, permeability, and structural organization (Tsuchiya, 2010). In grape leaves, anthocyanin accumulation under stress correlates with lower membrane peroxidation and improved bilayer stability (Pang et al., 2023), indicating that the protective effects of flavonoid depends on their subcellular localization as well as their antioxidant properties. Similarly, lanthanum-treated soybean seedlings showed reduced MDA accumulation and maintained plasma membrane permeability through enhanced scavenging of O_2_^-^ and ·OH radicals (Peng and Zhou, 2009). The halophyte *Sesuvium portulacastrum* further exemplifies this specialization through accumulation of highly methoxylated flavone glycosides associated with enhanced salinity tolerance (Wang et al., 2022a). The enrichment of methoxylated flavone glycosides further suggests that structural modifications influence membrane-associated functions, extending the role of flavonoids beyond radical scavenging to stabilization of stress-sensitive cellular structures.

Comparative studies under salinity and thermal stress further indicate that membrane protection depends on selective flavonoid reprogramming rather than uniform metabolic accumulation. Salt-tolerant cultivars generally maintain higher flavonoid levels together with reduced membrane injury relative to sensitive genotypes (Chutipaijit et al., 2009). Likewise, heat-induced ROS accumulation disrupts membrane fluidity and protein-lipid interactions, whereas flavonoid enrichment appears to preserve membrane-associated metabolic functions (Dos Santos et al., 2022; Saini et al., 2022). Tomato plants exposed to both heat and cold stress accumulate elevated catechin, rutin, and quercetin levels (Alhaithloul et al., 2021), suggesting that flavonoid-mediated membrane protection operates across contrasting thermal environments. These findings indicate that membrane protection depends not only on flavonoid abundance but also on the structural properties and localization of specific flavonoid subclasses. This spatial specialization broadens the role of flavonoids from ROS scavengers to regulators of membrane stability during abiotic stress.

### 5.4 Transcriptional and epigenetic reprogramming

Abiotic stress adaptation requires rapid transcriptional reprogramming that redirects metabolic resources from growth toward survival (Wang and Wang, 2026). Flavonoid biosynthesis constitutes a major component of this response; however, pathway activation is highly selective, temporally regulated and tightly integrated with broader stress-signaling networks (Akhtar et al., 2026). Consequently, stress resilience depends not merely on flavonoid accumulation but on the coordinated regulation, persistence, and tissue-specific deployment of flavonoid biosynthetic pathways, allowing plants to generate flavonoids profiles suited to changing environmental conditions.

Stress-induced activation of anthocyanidin synthase (OsANS) and dihydroflavonol 4-reductase (OsDFR) has been linked to regulation by the MYB transcription factor OsC1, suggesting a tightly controlled regulatory module rather than nonspecific pathway induction (Ithal and Reddy, 2004). Comparative studies in peanut, sesame, and wheat further reveal that tolerant genotypes sustain stronger and more persistent induction of flavonoid biosynthetic genes than sensitive cultivars (Yadav et al., 2021), indicating that stress resilience depends on regulatory durability rather than transient metabolic accumulation alone. These findings suggest that sustained transcriptional regulation, rather than transient pathway activation, is a distinguishing feature of stress-tolerant genotypes. Central regulation of flavonoid biosynthesis is mediated by MYB, bHLH, and WD40 transcription factor families (Schaart et al., 2013; Xu et al., 2015a). Coordinated expression of MYB transcription factors and flavonoid biosynthetic genes contributes to tissue-specific flavonoid accumulation patterns in plants (Liang et al., 2019). Moreover, conserved MYB- bHLH- WDR complexes function as higher-order transcriptional regulators that fine-tune flavonoid biosynthesis through coordinated and positive regulatory interactions (Xu et al., 2015a). By controlling the expression of branch-point biosynthetic genes, these regulatory complexes influence not only flavonoid abundance but also the relative production of different flavonoid subclasses, thereby shaping the structural diversity that underpins their stress-specific functions. Such multilayered regulation provides a mechanistic basis for the selective accumulation of distinct flavonoid subclasses, enabling plants to tailor flavonoid composition.

Epigenetic regulation adds an extra layer of control by modulating the long-term responsiveness of stress-associated pathways. Salt-primed soybean seedlings exhibit altered H3K4me2/H3K4me3 and H3K9ac enrichment at stress-responsive loci (Yung et al., 2022), whereas salinity-induced changes in siRNA accumulation and CHH methylation regulate *AtMYB74*-dependent ABA/ROS signaling in *Arabidopsis* (Xu et al., 2015b). Although direct chromatin-level regulation of flavonoid structural genes remains poorly understood, current evidence suggests that repeated stress exposure may progressively modify the inducibility of flavonoid biosynthetic pathways through epigenetic reprogramming. In *Arabidopsis*, repeated drought and heat stress induce persistent chromatin signatures, including H3K4 methylation and changes in nucleosome occupancy, that remain detectable after stress removal and facilitate subsequent activation of stress responsive genes (Ding et al., 2012; Lämke and Bäurle, 2017). Similar memory-associated mechanisms have been linked to improved acclimation under recurring abiotic stress conditions through the maintenance of transcriptionally competent chromatin states (Avramova, 2015; Friedrich et al., 2019). Whether comparable chromatin states directly regulate flavonoid structural genes remains unclear, but the available evidence supports an indirect role through upstream transcriptional regulators and stress-signaling pathways. Because flavonoid biosynthesis is regulated by ABA-responsive signaling, redox dependent pathways, and MYB-family transcription factors, epigenetic modifications affecting these regulatory components could influence the threshold and persistence of flavonoid pathway activation during repeated stress exposure (Xu et al., 2015a,b; Yung et al., 2022). The prolonged expression of flavonoid biosynthetic genes reported in several stress-tolerant genotypes (Yadav et al., 2021) is consistent with this hypothesis, although direct mechanistic links between stress memory and flavonoid-specific chromatin regulation remain to be established. Flavonoid biosynthesis should therefore be viewed as part of a multilayered regulatory network in which transcriptional control and epigenetic memory together influence the magnitude, timing, and persistence of flavonoid-mediated stress adaptation.

### 5.5 Osmotic adjustment and ion homeostasis, and metabolic coordination

Drought and salinity impose combined osmotic and ionic stresses that disrupt cellular water balance, membrane selectivity, and metabolic coordination (Gillani et al., 2022; Samanta et al., 2024; Hina et al., 2025b). Under these conditions, flavonoid-mediated adaptation extends beyond antioxidant defense to support osmotic buffering, ion homeostasis, and the metabolic adjustments required to maintain cellular function (Wagay et al., 2023; Rao and Zheng, 2025). Excessive Na^+^ accumulation during salinity destabilizes membranes and impairs enzymatic activity (Huang et al., 2024). In tolerant genotypes, enhanced flavonoid accumulation frequently coincides with increased proline biosynthesis and improved osmotic adjustment (Chutipaijit et al., 2009), suggesting coordinated regulation of osmotic and antioxidant metabolism during stress adaptation. Multi-omics analyses in *Setaria italica* further revealed coordinated enrichment of flavonoid, lignin, and phenylpropanoid pathways in slat-tolerant lines (Pan et al., 2020). This coordinated response suggests that osmotic adaptation relies on metabolic reprogramming rather than isolated ion transport. The simultaneous activation of these pathways suggests that flavonoid biosynthesis operates as part of broader metabolic network that balances structural reinforcement, osmotic adjustment, and redox protection under salinity stress.

The adaptive role of flavonoids also extends beyond intracellular protection to membrane stabilization and interactions within the rhizosphere (Álvarez‐Rodríguez et al., 2024). Flavonoids reduce lipid peroxidation and preserve membrane integrity under salinity stress (Zhou et al., 2023), while flavonoid accumulation in halophytes such as *Limonium bicolor* and *Sesuvium portulacastrum*, is associated with exceptional ionic tolerance and oxidative resilience (Nikalje et al., 2018). Elevated levels of ferulic acid, caffeic acid, quercetin, and kaempferol in salt-tolerant plants further support stress-associated redox stability and membrane protection (Kiani et al., 2024). These observations indicate that flavonoids contribute to osmotic adaptation by coordinating redox homeostasis, membrane stability, and metabolic reprogramming rather than functioning solely as antioxidants. Their integration with broader phenylpropanoid networks further highlights their role in maintaining cellular homeostasis under salinity and drought stress.

### 5.6 Phytohormone signaling modulation

Flavonoid-mediated abiotic stress adaptation is closely integrated with phytohormone signaling pathways that coordinate plant growth, development, and stress responses (Akhtar et al., 2026). Beyond their antioxidant functions, flavonoids influence hormonal sensitivity, transport, and downstream signaling, thereby contributing to stress-induced developmental reprogramming (Akhtar et al., 2010). These regulatory interactions enable plants to adjust growth patterns while maintaining physiological resilience under adverse environmental conditions. Among phytohormones, the relationship between flavonoids and auxin signaling is particularly well established. Stress-induced flavonol accumulation can modulate auxin transport through effects on auxin efflux carriers, resulting in altered auxin distribution within plant tissues (Lewis et al., 2011). Such changes influence root architecture, cell elongation, and developmental plasticity, allowing plants to optimize resource acquisition and growth under stress. The reciprocal relationship between auxin signaling and flavonoid biosynthesis indicates that flavonoid function not only as metabolic products but also as regulators of developmental plasticity during stress adaptation (Akhtar et al., 2010).

Flavonoid metabolism is also closely associated with abscisic acid (ABA)-dependent stress responses. During drought and salinity stress, ABA accumulation promotes the activation of phenylpropanoid metabolism and the biosynthesis of protective secondary metabolites, including flavonoids. Because ABA-responsive transcription factors also regulate key flavonoid biosynthetic genes, hormonal signaling influences not only pathway activation but also the composition of flavonoids produced under stress. In rice, *OsOLP-*mediated drought tolerance involves ABA-responsive regulation of phenylpropanoid-associated pathways, highlighting the integration of flavonoid metabolism within broader hormonal adaptation mechanisms (Yan et al., 2023). These interactions position flavonoids at the interface of hormonal signaling and metabolic regulation, coordinating growth restraint with stress acclimation. The influence of flavonoids on phytohormone signaling is highly dependent on stress intensity, developmental stage, and tissue type. Rather than acting through maximal accumulation, flavonoids fine-tune hormonal responsiveness to balance growth with stress adaptation.

Collectively, the studies discussed in this section demonstrate that flavonoid-mediated abiotic stress adaptation is governed by regulatory flexibility rather than fixed metabolic outputs. As summarized in **Table 3**, individual stresses recruit both shared and specialized flavonoid responses, reflecting a balance between conserved protective functions and stress-specific metabolic requirements. However, because these regulatory programs rarely operate in isolation under natural environments, understanding their points of convergence, interaction, and prioritization becomes essential for explaining plant performance under realistic stress conditions. This shift from stress-specific responses to regulatory integration provides the basis for examining flavonoid reprogramming under combined stress.

**Table 3:** Comparative roles of flavonoid subclasses and regulatory pathways across major abiotic stresses.

Stress	Flavonoid subclasses	Major regulatory pathways	Responses	Plant species	References
Drought	Flavonols (quercetin, kaempferol), catechins, anthocyanins	ABA signalling, ROS-scavenging pathways, MYB–bHLH transcriptional regulation	ROS detoxification, osmotic adjustment, drought acclimation	*Arabidopsis thaliana*, *Sesamum indicum, Camellia sinensis*	(Lv et al., 2021; Nakabayashi et al., 2014; You et al., 2019)
Salinity	Flavonols, flavone glycosides, methoxylated flavones	ABA signalling, antioxidant defense pathways, ion-homeostasis regulation	ROS mitigation, membrane protection, salt tolerance	*Glycine max*, *Sesuvium portulacastrum*	(Nikalje et al., 2018; Pi et al., 2023)
Heat	Flavonols, isoflavones, anthocyanins	Heat-shock factor (HSF) signalling, ROS-responsive pathways	Antioxidant protection, maintenance of photosynthetic function, heat tolerance	Glycine max, Solanum lycopersicum	(Chebrolu et al., 2016; Postiglione et al., 2024)
UV-B	Quercetin glycosides, kaempferol glycosides, luteolin derivatives, anthocyanins	UVR8–COP1–HY5 signalling, R2R3-MYB transcription factors	UV screening, photoprotection, antioxidant defense	Arabidopsis thaliana, Vitis vinifera	(Berli et al., 2011; Neugart et al., 2024)

## 6 Convergent regulatory reprogramming of flavonoids under combined stress

**Figure 4:**
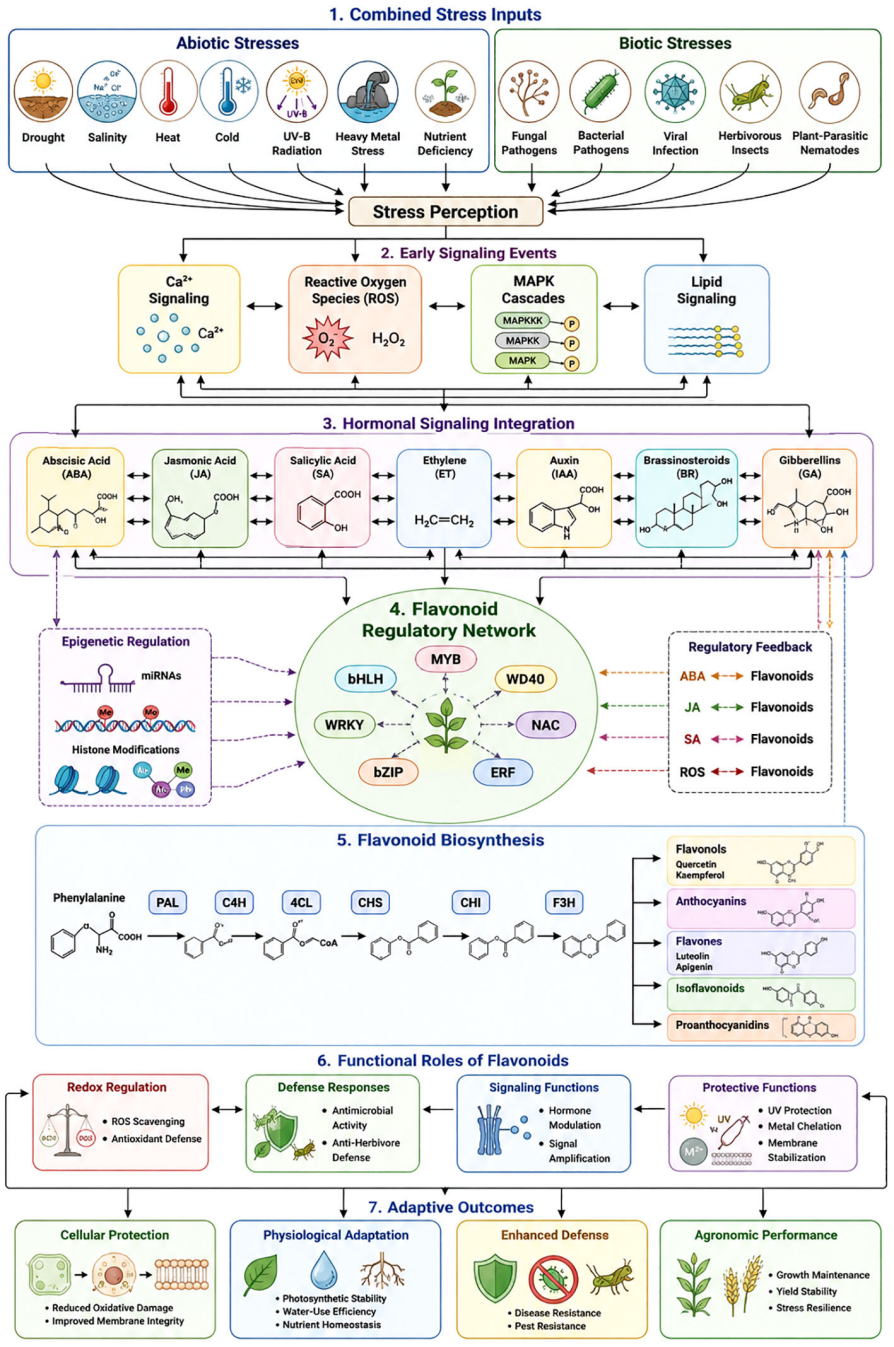
Flavonoid-centered signaling networks integrating plant responses to combined abiotic and biotic stresses. Combined abiotic (drought, salinity, heat, cold, UV-B radiation, heavy metals, and nutrient deficiency) and biotic stresses (pathogens, insects, viruses, and nematodes) trigger stress perception and early signaling events involving Ca²^+^, ROS, MAPK cascades, and lipid signaling. These signals interact with phytohormone pathways (ABA, JA, SA, ET, auxin, BR, and GA) and converge on a flavonoid regulatory network controlled by transcription factors and epigenetic mechanisms. Flavonoid biosynthesis generates diverse metabolites that contribute to antioxidant defense, stress signaling, antimicrobial protection, UV shielding, metal chelation, and membrane stabilization. Feedback interactions between flavonoids and ROS/hormonal pathways enhance stress adaptation, ultimately promoting cellular protection, physiological resilience, enhanced defense, and stable plant growth under combined stress conditions.

Combined environmental stresses impose greater constraints on plant growth and productivity than individual stressors alone (Jumrani and Bhatia, 2018). Plant responses to stress combinations are not merely additive extensions of single-stress acclimation. Instead, combined stresses are perceived as distinct physiological states that trigger coordinated metabolic, transcriptional, and signaling reprogramming involving redox balance, hormonal crosstalk, carbon allocation, and defense prioritization (Pandey et al., 2015; Sánchez-Bermúdez et al., 2022; Priya et al., 2023; Jiang et al., 2026) (**Figure 4**). These responses indicate that the phenylpropanoid pathway operates not as a linear defense system but as a flexible, reconfigurable metabolic network. This regulatory plasticity likely contributes to the inconsistent field performance of many flavonoid-based engineering strategies across environments and reinforces the need for systems-level approaches. The following sections examine how convergent redox and hormonal signaling, non-linear phenylpropanoid reprogramming, metabolic-trade-offs, spatiotemporal plasticity, and pathway-level regulatory constraints shape the opportunities and limitations of predictive flavonoid engineering.

### 6.1 Shared redox and hormonal signaling hubs under combined stress

Plant responses to combined stresses are coordinated through interconnected reactive oxygen species (ROS), calcium, mitogen-activated protein kinase (MAPK), and hormonal signaling networks, yet these responses rarely represent simple combinations of those observed under individual stresses (Atkinson et al., 2013; Prasch and Sonnewald, 2013; Suzuki et al., 2012; Xiong and Yang, 2003). Within these networks, flavonoids function not only as antioxidants, but also as modulators of redox homeostasis, hormone sensitivity, and stress signaling. By buffering ROS accumulation and influencing downstream signaling cascades, flavonoids contribute to the integration of environmental cues and the coordination of defense responses (Ku et al., 2018).

Under combined stress, extensive crosstalk among salicylic acid (SA), jasmonic acid (JA), abscisic acid (ABA), ethylene, and auxin generates regulatory interactions that are often antagonistic or conditional on the stress combination (Zandalinas et al., 2021). ABA and JA are central to osmotic and ionic stress adaptation, whereas auxin and ethylene contribute to developmental plasticity and growth-defense trade-offs (Riemann et al., 2015). At the same time, ABA frequently suppresses SA- and JA/ET-mediated immune pathways, creating a regulatory interface between abiotic acclimation and pathogen defense (Kissoudis et al., 2014; Shigenaga et al., 2017). Flavonoids participate in this interface by modulating auxin transport, influencing root architectural responses, and contributing to the maintenance of cellular redox balance under stress (Li et al., 2018).

Salinity stress provides a representative example of this signaling convergence. Elevated ROS levels and ABA signaling coordinately activate antioxidant systems and the phenylpropanoid-flavonoid pathway, promoting the accumulation of flavonols and anthocyanins that preserve photosynthetic integrity and membrane stability (Zhao et al., 2021b). Beyond their protective functions, flavonoids can influence the activity of ABA-responsive regulators, including DREB, AREB/ABF, MYB, and MYC transcription factors, thereby establishing feedback interactions between metabolic and signaling networks (Crizel et al., 2020; Hussain et al., 2021). Many of these hormonal and redox signals converge on MYB–bHLH–WD40 (MBW) regulatory complexes that control the expression of key flavonoid biosynthetic genes, linking environmental perception directly to flavonoid pathway reprogramming (Schaart et al., 2013). Because MBW complexes integrate inputs from multiple signaling pathways, their regulatory output can vary substantially between individual and combined stresses, even when similar upstream signals are activated.

The complexity of this regulation increases further when abiotic and biotic stresses occur simultaneously. ROS generated during drought, salinity, or heat stress also serve as essential signaling molecules during pathogen attack and herbivory, creating shared signaling hubs that integrate acclimation and defense responses (Haraguchi et al., 2000; Rackova et al., 2005; Samanta et al., 2011). Transcriptomic analyses further show that flavonoid regulation under combined stresses frequently induce gene-expression profiles that cannot be predicted from individual stress responses. For example, drought-ozone and drought-heat combinations trigger distinct transcriptional programs involving hormonal signaling, redox regulation, and secondary metabolism, indicating extensive regulatory rewiring under multifactorial stress conditions (Choi et al., 2013; Iyer et al., 2013). These observations indicate that flavonoid accumulation under combined stress is governed by the integration of redox, hormonal, and transcriptional regulatory networks rather than by antioxidant demand alone. Consequently, similar environmental signals can produce different flavonoid outputs depending on the accompanying stress, illustrating that pathway regulation is determined by network integration rather than by individual signaling pathways. This network-level behavior lays the foundation for broader reconfiguration of phenylpropanoid metabolism under combined stress.

### 6.2 Non-linear reconfiguration of phenylpropanoid and flavonoid flux

Combined stresses substantially alter carbon allocation and secondary metabolic flux, resulting in phenylpropanoid and flavonoid responses that differ from those observed under individual stress conditions (Mittler, 2006; Mittler and Blumwald, 2010). Transcriptomic analyses indicate that only a limited proportion of stress-responsive genes exhibit additive regulation during stress combinations, whereas most display synergistic, antagonistic, or unique expression patterns (Rizhsky et al., 2004; Rasmussen et al., 2013). This non-linearity arises because multiple redox, hormonal, and metabolic signaling pathways converge on shared transcriptional regulators, biosynthetic enzymes, and precursor pools, redistributing metabolic flux among competing phenylpropanoid outputs rather than uniformly increasing activity across the pathway (Atkinson et al., 2013). Consequently, flavonoid accumulation under combined stress reflects the balance between competing defense and acclimation priorities rather than direct activation of a single biosynthetic branch.

Because the phenylpropanoid-flavonoid pathway occupies a central position at the interface of stress signaling and carbon metabolism, its metabolic output is highly sensitive to stress convergence. Under UV-B or excess irradiance, enhanced flavonol biosynthesis primarily contributes to photoprotection and ROS scavenging (Patil et al., 2024). However, when biotic stress signals are simultaneously activated, phenylpropanoid flux may be redirected toward lignification, phytoalexin biosynthesis, and other immune-associated metabolites (Schenke et al., 2019). This reallocation is evident during UV-B/flg22 interactions, where UV-B-induced flavonol accumulation is suppressed following flg22-mediated immune activation, accompanied by increased production of camalexin, scopoletin, and lignin-associated compounds through antagonistic regulation involving MYB12 and MYB4 transcription factors (Logemann and Hahlbrock, 2002; Schenke et al., 2011). These observations indicate that phenylpropanoid metabolism under combined stress is governed by selective redistribution of metabolic resources rather than uniform pathway induction. Consequently, the accumulation of individual flavonoids cannot be interpreted independently of the competing metabolic demands imposed elsewhere in the phenylpropanoid network.

Metabolic competition also occurs within the flavonoid pathway itself. Branch-point enzymes such as flavonol synthase (FLS), dihydroflavonol 4-reductase (DFR), anthocyanidin synthase (ANS), anthocyanidin reductase (ANR), and leucoanthocyanidin reductase (LAR) compete for common intermediates, thereby influencing the relative accumulation of flavonols, anthocyanins, and proanthocyanidins (Lei et al., 2023). Under combined stress conditions, changes in transcription factor activity, hormone signaling, or precursor availability can shift carbon flux among these competing branches, generating metabolic outcomes that are difficult to predict from single-stress responses alone (Zandalinas et al., 2021). Branch-point competition therefore represents a major source of phenylpropanoid pathway plasticity and contributes to the emergence of stress-specific flavonoid profiles.

The direction and magnitude of flux redistribution are further influenced by genetic background. In *Solanum lycopersicum*, combined heat and salinity stress enhanced flavonoid accumulation through induction of F3H expression, whereas salinity alone suppressed F3H transcripts (Martinez et al., 2016). In contrast, salinity stress increased F3H expression in *Reaumuria trigyna* (Zhang et al., 2014). These contrasting responses indicate that flavonoid reprogramming under combined stress depends not only on stress composition but also on species-specific regulatory architectures and metabolic capacities, limiting the transferability of engineering strategies across species (Chaturvedi et al., 2024).

Combined stress additionally reshapes primary carbon metabolism, thereby influencing substrate availability for phenylpropanoid biosynthesis. Reduced photosynthetic efficiency and altered mitochondrial function increase glycolytic flux and soluble sugar accumulation to sustain ATP production and osmotic adjustment (ElSayed et al., 2022; Roychoudhury, 2022). Multi-omics analyses suggest that part of this redirected carbon is channeled into phenylpropanoid and flavonoid biosynthesis, supporting ROS detoxification, ascorbate–glutathione cycling, and redox stabilization (Nadarajah, 2020; Waszczak et al., 2024). Hormonal signals further influence this metabolic reallocation. For example, methyl jasmonate treatment under salinity stress in *Ginkgo biloba* enhanced the expression of flavonoid biosynthetic genes, increased quercetin accumulation, and reduced oxidative damage, illustrating how stress-responsive signaling pathways can redirect metabolic resources toward flavonoid production (Feng et al., 2025). Similarly, salinity and ABA signaling frequently induce the expression of PAL, CHS, F3H, FLS, ANS, and UDP-glucose:flavonoid 3-O-glucosyltransferase (UFGT), promoting the accumulation of flavonoids and phenolic acids associated with osmotic adjustment and redox homeostasis (Skodra et al., 2021). Overall, these findings demonstrate that phenylpropanoid and flavonoid metabolism under combined stress is continuously reconfigured through the redistribution of metabolic flux across competing defense, acclimation, and developmental pathways. As a result, flavonoid accumulation reflects the integration of multiple regulatory and metabolic priorities rather than the activation of a single biosynthetic program. This network-level complexity presents a major challenge for predictive modelling and provides the rationale for approaches capable of resolving regulatory interactions across the entire phenylpropanoid network.

### 6.3 Metabolic trade-offs and defense prioritization

Combined biotic and abiotic stresses impose simultaneous and often conflicting physiological demands on plants, forcing them to continuously balance resource allocation among growth, maintenance, reproduction, and defense (Pandey et al., 2017). Although flavonoids contribute to stress protection through antioxidant activity, signaling modulation, and defense functions, their biosynthesis requires substantial investments of adenosine triphosphate (ATP), reducing equivalents, phenylalanine, acetyl-CoA, and malonyl-CoA that could otherwise support primary metabolism, respiration, osmotic adjustment, or reproductive development (Dixon and Paiva, 1995; Huot et al., 2014). Under prolonged or multifactorial stress conditions, plants cannot maximize all protective responses simultaneously but must prioritize metabolic investments according to the nature and severity of the prevailing stresses (Mittler, 2006; Zandalinas et al., 2021). Metabolomic and physiological studies suggest that combined stress restructures carbohydrate, amino acid, and tricarboxylic acid (TCA) cycle metabolism to sustain essential defense and acclimation processes under conditions of limited energy availability (Garcia-Molina and Pastor, 2024). Consequently, successful stress adaptation depends on how efficiently metabolic resources are redistributed rather than on constitutively elevated flavonoid accumulation. This prioritization becomes particularly important when stress combinations simultaneously require osmotic adjustment, antioxidant protections, immune activation, and maintenance of cellular homeostasis. In such situations, investment in one defense process may occur at the expense of another, reflecting coordinated prioritization rather than maximal activation of all protective pathways.

Defense prioritization also extends across the broader phenylpropanoid network. During pathogen attack, carbon resources are often redirected toward lignin deposition, phytoalexin synthesis, and cell wall reinforcement pathways that provide rapid structural and antimicrobial protection (Dixon and Paiva, 1995). For example, UV-B-induced flavonol accumulation can be suppressed during immune activation through antagonistic MYB regulation, redirecting metabolic resources toward defense-associated phenolics and antimicrobial compounds (Logemann and Hahlbrock, 2002; Schenke et al., 2011). Similar occur under combined stress, where maintenance of lignification and pathogen defense responses may occur at the expense of flavonoid diversification, growth-related metabolism, or reproductive investment (Muro-Villanueva et al., 2019). These shifts indicate that metabolic competition operates across the entire phenylpropanoid network rather than within individual flavonoid branches alone.

Trade-offs also emerge from differences in the ecological functions of individual flavonoid metabolites. Because flavonoid subclasses vary in their effects on herbivores, pathogens, and beneficial interactions, increased flavonoid accumulation does not necessarily translate into enhanced stress resistance. For instance, MYB12-mediated flavonol accumulation improves resistance against *Spodoptera litura* and *Helicoverpa armigera* (Misra et al., 2010), whereas MYB75-associated accumulation of kaempferol-3,7-dirhamnoside unexpectedly increases susceptibility to *Pieris brassicae* (Onkokesung et al., 2014). These contrasting outcomes illustrate that defense effectiveness depends not only on the quantity of flavonoids produced but also on their composition, localization, and ecological function.

Resource limitations become particularly pronounced during prolonged stress exposure and reproductive development, when secondary metabolism competes directly with growth and yield-associated sink demands (Westgate, 1994; Barnabás et al., 2008). Consequently, optimal stress adaptation relies on coordinated allocation of metabolic resources among competing defense, acclimation, and developmental processes rather than maximal activation of flavonoid biosynthesis. These trade-offs represent a fundamental constraint because enhancing one defense function may inadvertently compromise growth, productivity, or resilience to other environmental challenges. Consequently, metabolic prioritization is expressed differently across biological contexts, adding another layer of complexity to flavonoid regulation under combined stress.

### 6.4 Spatial and temporal plasticity of flavonoid reprogramming

The protective functions of flavonoids under combined stress depend not only on their abundance but also on where and when they accumulate. Consequently, flavonoid responses frequently differ among organs, tissues, cell types, and developmental stages, patterns that are often obscured by whole-tissue analyses. Current evidence suggests that flavonoid-mediated adaptation relies on selective deployment to sites of greatest physiological demand rather than uniform accumulation throughout the plant (Tattini et al., 2000; Ahad et al., 2026).

Spatial organization plays a central role in determining flavonoid function under stress. In *Arabidopsis thaliana* exposed to combined high light and heat stress, anthocyanins accumulate preferentially in photosynthetically active leaf tissues experiencing elevated oxidative pressure, indicating that flavonoid deposition is coordinated with local physiological requirements rather than occurring uniformly throughout the plant (Balfagón et al., 2019). Organ-specific responses have also been observed in tomato, where heat and salinity combinations produce distinct metabolic signatures in leaves and roots, reflecting differences in photosynthetic activity, ion regulation, and environmental exposure (Martinez et al., 2016). Beyond organ-level specialization, flavonoids are differentially compartmentalized within cells. Vacuolar sequestration facilitates long-term storage and detoxification, whereas accumulation in epidermal tissues and chloroplast-associated regions provides localized protection against excess radiation and oxidative damage. Such compartmentalization enables plants to deploy flavonoids where protection is most urgently required while minimizing unnecessary metabolic expenditure (Agati et al., 2012; Nakabayashi et al., 2014).

Spatial plasticity also extends beyond plant tissues into the rhizosphere. Flavonoids released from roots influence microbial recruitment, nutrient acquisition, and stress-associated plant-microbe interactions, thereby contributing to environmental adaptation through mechanisms that extend beyond intracellular antioxidant functions (Kumar et al., 2024). Experimental studies further demonstrate that exogenous flavonoids such as naringenin and rutin can enhance tolerance to ionic and oxidative stress by modulating antioxidant capacity, osmotic adjustment, and photosynthetic performance (Yildiztugay et al., 2020). These observations highlight the diverse spatial scales at which flavonoids operate, ranging from subcellular protection to ecosystem-level interactions.

Temporal regulation adds a further level of complexity. Flavonoid profiles often change throughout stress exposure, with different metabolites accumulating during stress perception, acclimation, and recovery. Consequently, responses observed at a single time point may provide only a partial view of the underlying metabolic adjustments. Sequential and simultaneous stress combinations can also produce distinct outcomes because prior stress exposure modifies subsequent transcriptional, metabolic, and physiological responsiveness (Bruce et al., 2007). In chickpea, for example, combined drought and *Ralstonia solanacearum* infection generated markedly different metabolic and transcriptomic responses during early and prolonged stages of stress exposure, demonstrating that defense-associated metabolism is strongly influenced by stress duration and order of occurrence (Sinha et al., 2017). Similar temporal shifts have been observed in broader metabolomic networks, where changes in flavonoids occur alongside coordinated adjustments in amino acid, carbohydrate, and respiratory metabolism during environmental stress adaptation (Garcia-Molina and Pastor, 2024).

Despite growing recognition of spatial and temporal heterogeneity under combined abiotic stresses, understanding of simultaneous biotic-abiotic stress remains comparatively limited. Most studies continue to rely on bulk tissue analyses that overlook cell-type-specific, tissue- and developmental-stage-specific patterns of flavonoid accumulation. Advances in spatial transcriptomics, single-cell profiling, and tissue-resolved metabolomics are beginning to address these limitations by revealing regulatory mechanisms that cannot be resolved using bulk analyses. Current evidence indicates that flavonoid-mediated adaptation depends not only on the magnitude of metabolic accumulation but also on its precise spatial and temporal deployment. This additional layer of regulation further constrains flavonoid reprogramming under combined stress, highlighting the importance of understanding how regulatory networks coordinate these responses across multiple biological levels.

### 6.5 Regulatory and systems-level constraints in flavonoid networks

Despite their extensive metabolic plasticity, flavonoid networks operate within a highly constrained regulatory architecture. The ability of plants to adjust flavonoid composition under combined stresses is therefore not unlimited but is restricted by hierarchical transcriptional control, competition for shared metabolic resources, and multiple layers of regulatory buffering. These constraints decouple transcriptional activation from metabolic output. Consequently, changes in gene expression do not always translate into predictable metabolic or phenotypic outcomes under combined stress making (Tohge et al., 2017; Yonekura-Sakakibara et al., 2019).

A central bottlenecks is the MYB–bHLH–WD40 (MBW) complex, which coordinates the expression of key flavonoid biosynthetic genes, including CHS, CHI, F3H, DFR, ANS, and UFGT (Hichri et al., 2011). Because MBW complexes occupy an upstream regulatory position, changes in their activity can simultaneously influence multiple branches of flavonoid metabolism. Consequently, manipulation of individual MYB or bHLH regulators frequently produces broader phenotypic effects than anticipated. For example, overexpression of VvMYB5b increases anthocyanin and proanthocyanidins accumulation but also alters broader phenylpropanoid metabolism and developmental processes (Deluc et al., 2008). Likewise, PAP1/MYB75 promotes anthocyanin biosynthesis yet can be associated with growth penalties and metabolic imbalances under certain stress environments (Borevitz et al., 2000). Although enhanced bHLH activity has been linked with improved salinity and drought tolerance in transgenic *Arabidopsis thaliana* (Wang et al., 2016), these studies collectively demonstrate that upstream regulatory nodes rarely control a single metabolic outcome. Instead, they influence interconnected processes whose responses depend on the broader regulatory context (Liu et al., 2015). Consequently, the same regulatory intervention can produce different phenotypic outcomes depending on the surrounding signaling environment, indicating that MBW complexes function as integrative regulatory hubs rather than simple transcriptional switches.

Further constraints arise from the organization of the flavonoid network. Because flavonoid biosynthesis is integrated within broader phenylpropanoid system (Tian et al., 2015; Luo et al., 2016; Lei et al., 2023), perturbation of a single pathways component can influence multiple metabolic outputs simultaneously. Consequently, metabolic behavior emerges from interactions among multiple pathway components, so altering a single enzyme rarely produces proportional or predictable changes in flavonoid composition. This complexity is reinforced by evidence for metabolon organization, where enzyme interactions, substrate channeling, and intracellular compartmentalization contribute to pathway behavior (Winkel, 2004). As a result, pathway architecture constrains engineering outcomes because regulatory interactions frequently override the effects of individual genetic modifications.

Flavonoid pathway behavior is also shaped by regulatory mechanisms that buffer transcriptional and metabolic responses. Histone modification, DNA methylation, chromatin remodeling, and microRNA-mediated regulation contribute to stress-responsive control of phenylpropanoid genes (Olejniczak et al., 2018). Changes in chromatin accessibility can alter the recruitment of transcription factors to biosynthetic loci and thereby influence the magnitude and persistence of pathway activation (Maher et al., 2018; Méteignier et al., 2023; Kamal and Shabbir, 2025). Histone acetylation and methylation have similarly been implicated in the activation and repression of secondary metabolic pathways (Hu et al., 2019), while enhancer-associated elements contribute additional layers of transcriptional regulation (Oka et al., 2017). Beyond chromatin-level control, post-regulational modifications such as phosphorylation, ubiquitination, and SUMOylation affect the stability and activity of MYB, bHLH, and WD40, thereby influencing MBW complex assembly and downstream pathway behavior (Li, 2014). Stress-associated chromatin modifications may also influence subsequent transcriptional responsiveness during prolonged or repeated stress exposure (Crisp et al., 2016; Lämke and Bäurle, 2017), whereas microRNAs targeting MYB transcription factors and anthocyanin-associated genes introduce additional variability into pathway activity (Gou et al., 2011). Together, these regulatory layers buffers transcriptional fluctuations while preserving metabolic stability. Although such buffering enhances stress resilience, it also reduces the predictability of engineering interventions because identical genetic modifications may produce different metabolic outputs under different environmental conditions (Escrich et al., 2022).

These constraints become increasingly apparent when flavonoid-associated traits are evaluated beyond controlled experimental conditions. Response that appears beneficial under one condition may become neutral, inconsistent, or even detrimental under different stress combinations or developmental stages (Mittler and Blumwald, 2010; Atkinson et al., 2013). Moreover, many mechanistic and engineering studies are conducted under highly controlled conditions that do not capture the temporal variability and environmental heterogeneity encountered in agricultural systems (Foyer et al., 2016). Under field conditions, genotype x environment x microbiome interactions can substantially alter metabolic composition, transport dynamics, and stress responsiveness, reducing the stability of flavonoid-associated phenotypes across locations and seasons (Vives-Peris et al., 2020; Farid et al., 2025). Consequently, increasing flavonoid accumulation alone is not a reliable predictor of improved stress resilience, yield stability, or agronomic performance because regulatory coordination, metabolic allocation, and environmental context ultimately determine phenotypic performance. These observations indicate that the principal challenge is no longer identifying additional flavonoid-associated genes or metabolites, but determining which regulatory interactions consistently govern metabolic behavior across diverse stress combinations. Distinguishing causal regulators from downstream responses remains particularly difficult because multiple signaling, metabolic, and developmental processes operate simultaneously. Resolving these interconnected relationships requires analytical frameworks capable of integrating information across entire regulatory networks rather than evaluating pathway components in isolation.

## 7 Artificial intelligence-assisted discovery and multi-omics decoding of flavonoid stress networks

Artificial intelligence is transforming how flavonoid metabolism is analyzed under complex stress environments. Conventional engineering strategies have largely focused on individual structural genes or metabolites (Khan et al., 2025; Tian et al., 2026). However, the complexity of flavonoid responses limits the predictive value of reductionist approaches because changes in individual genes rarely explain network-level behavior. Computational frameworks that integrate large-scale biological datasets therefore provide a more suitable basis for identifying regulatory relationships that can be tested experimentally. Recent advances in machine learning and multiomics integration have substantially improved reconstruction of flavonoid regulatory networks. ML algorithms integrate genomic, transcriptomic, metabolomic, proteomic, and environmental datasets to identify enzyme functions, pathway membership, transcriptional coordination, and metabolic bottlenecks within phenylpropanoid metabolism (Singh et al., 2016; Silva et al., 2019). In *Arabidopsis thaliana*, AI-based frameworks improved the prediction of metabolic pathways associations and enzyme functions, leading to more accurate annotation of specialized metabolism (Bai et al., 2024). Similarly, the AI-Ready Cross-Scale Transcriptome Integration and Correlation Analysis (ARCTICA) framework demonstrated that combining ML with genome-scale metabolic modeling can identify and manipulate metabolic fluxes, providing a foundation for crop metabolic engineering (Kugler and Stensjö, 2024). These approaches are particularly valuable under combined stress, where flavonoid accumulation reflects coordinated reorganization of interconnected metabolic and signaling pathways rather than linear transcriptional responses (Kissoudis et al., 2014).

### 7.1 AI-assisted integration of multi-omics for flavonoid network reconstruction

Flavonoid biosynthesis is regulated through interconnected layers of chromatin organization, transcriptional control, protein interactions, metabolic transport, and environmental signaling (Ren et al., 2026). Consequently, individual omics approaches provide only fragmented views of pathway regulation, whereas integrating transcriptomic, metabolomic, proteomic, and genomic datasets enables comprehensive reconstruction of flavonoid-associated regulatory networks (Shah et al., 2021). Integrating multiple omics layers increases biological resolution but also generates high-dimensional datasets that are difficult to interpret using conventional statistical approaches. AI address this challenge by identifying non-linear relationships and coordinated regulatory modules that may not be apparent when individual datasets are analyzed separately. This capability is particularly valuable under combined stress, where interacting biotic and abiotic signals generate regulatory states that cannot be inferred from individual omics layers or single stress analyses.

Recent machine learning approaches have accelerated identification of specialized metabolic genes and regulatory modules associated with phenylpropanoid and flavonoid metabolism. By integrating co-expression profiles (Uygun et al., 2016), subcellular co-localization (Davis et al., 2015), and biosynthetic gene cluster analyses (Nützmann et al., 2016), ML models have improved identification of genes involved in specialized metabolism across diverse plant species. For example, ML frameworks successfully distinguished primary and specialized metabolic genes using genomic and biochemical characteristics (Moore et al., 2019), while pathway-associated genes were accurately predicted in tomato through integrative ML models (Wang et al., 2021). Similarly, multi-omics models developed in *Arabidopsis* identified genes associated with specialized metabolite biosynthesis with high accuracy and demonstrated transferability to other crop species (Jumper et al., 2021; Akagi et al., 2022). Although most existing models have been developed using single-stress or non-stress datasets, their ability to integrate diverse molecular features provides a framework for reconstructing regulatory networks under combined stress, where pathway interactions become substantially more complex.

Beyond gene discovery, integrating environmental metadata with multi-omics datasets is improving understanding of how flavonoid regulatory networks respond to changing stress environments. Rather than focusing solely on individual stress factors, these models can identify regulatory states associated with adaptive resilience under multiple environmental constraints (Farooq et al., 2024; Iqbal et al., 2024). Under simultaneous biotic and abiotic stresses, however, regulatory interactions become highly non-linear. As noted earlier, signaling pathways, metabolic fluxes, and developmental responses influence one another. AI is particularly suited to disentangling these interconnected responses by analyzing multiple molecular layers simultaneously. Emerging deep-learning frameworks are also being used to identify how key transcription factors, including MYB, WRKY, and bZIP proteins, influence downstream flavonoid accumulation and metabolic reprogramming under stress conditions (Chen et al., 2026a). Together these advances extend AI applications beyond candidate gene discovery towards reconstruction of regulatory hierarchies that govern flavonoid responses under complex stress environment. However, most current models are trained using datasets generated under individual stress conditions, limiting their ability to resolve the regulatory interactions that emerge specifically under combined biotic and abiotic stresses.

### 7.2 Machine learning for functional prediction and metabolic optimization

AI-assisted functional prediction offers new opportunities to advance flavonoid engineering beyond simple pathway activation towards the prediction and optimization of stress-adaptive metabolic outputs. Conventional engineering strategies often target individual biosynthetic genes, yet flavonoid accumulation is constrained by precursor availability, carbon allocation, transport processes, and regulatory feedback mechanisms that collectively influence pathway performance (Jiang et al., 2018; Zhang et al., 2025). As a result, increasing flavonoid production does not necessarily translate into improved stress tolerance and may impose fitness or growth penalties under certain conditions.

Machine learning approaches are particularly valuable for identifying the complex interactions that govern flavonoid metabolism. By integrating transcriptomic, metabolomic, and phenotypic datasets, ML models can identify regulatory relationships linking gene activity with branch-specific metabolite accumulation and stress-associated traits (Lo-Thong-Viramoutou et al., 2022). Such analyses provide insights into how metabolic flux is distributed among competing flavonoid branches, including flavonols, anthocyanins, and phytoalexins, thereby facilitating prediction of pathway responses to genetic or environmental perturbations (Song and Ramkrishna, 2013). Although these predictive relationships are increasingly robust, their application to flavonoid engineering under combined stress remains limited by the availability of integrated training datasets representing multiple environmental scenarios.

Integration of ML with genome-scale metabolic modelling further strengthens predictive capabilities by linking flavonoid biosynthesis with whole-plant carbon economy and metabolic resource allocation (Cheung et al., 2014). These approaches have demonstrated that stress adaptation frequently depends on selective enhancement of specific flavonoid subclasses rather than uniform activation of the entire phenylpropanoid pathway (Wang et al., 2025). Consequently, AI-guided multigene engineering strategies informed by metabolic flux dynamics may provide more stable outcomes than single-gene interventions, which can disrupt pathway equilibrium and trigger compensatory responses (Xu et al., 2016).

Recent advances in high-throughput phenotyping and remote sensing are further expanding the predictive capacity of AI-assisted metabolic engineering. Integration of ML algorithms with unmanned aerial vehicle (UAV)-based hyperspectral imaging (HSI) enables large-scale association of stress-responsive phenotypes with underlying metabolic reprogramming (Costa et al., 2022; Gill et al., 2022). However, because flavonoid accumulation is highly tissue- and cell-specific, future progress will require closer integration of phenotyping platforms with spatial metabolomics and single-cell omics to improve biological interpretations and predictive accuracy (Mmbando, 2024). Collectively, these developments provide a framework for moving flavonoid engineering beyond empirical gene manipulation towards predictive optimization of metabolic fluxes. As these approaches mature and are validated under combined stress conditions, they may facilitate the identification of intervention strategies that enhance resilience while minimizing adverse effects on plant growth and development.

### 7.3 Network biology and dynamic system modelling

While multi-omics integration can identify genes, metabolites, and pathways associated with flavonoid accumulation, understanding how these components interact requires network-based modelling approaches. Such frameworks provide insight into regulatory connectivity, metabolic constraints, and temporal responses that collectively determine flavonoid reprogramming under stress conditions. Gene co-expression analysis has become an important strategy for identifying transcriptionally coordinated modules associated with flavonoid biosynthesis and stress adaptation. Among available approaches, Weighted Gene Co-expression Network Analysis (WGCNA) is widely used to detect highly connected hub genes linked to specialized metabolism (Langfelder and Horvath, 2008). In several plant species, co-expression networks have associated MYB, bHLH, WD40, and NAC transcription factors with branch-specific flavonoid accumulation patterns and stress-responsive phenylpropanoid reprogramming (Ma et al., 2014; Xu et al., 2015a). Although co-expression relationships do not establish causality, integrating module connectivity, hub-gene centrality, and network topology with ML frameworks improves the prioritization of candidate regulators for experimental validation and metabolic engineering (Badia-i-Mompel et al., 2023). Such analyses are particularly useful for distinguishing central regulatory hubs from downstream transcriptional responses that emerge during stress exposure. This distinction is particularly important for engineering because modifying downstream responsive genes often produce transient phenotypic effects, whereas manipulation of upstream regulatory hubs is more likely to generate coordinated and durable metabolic responses.

Beyond co-expression, understanding how flavonoid regulatory hierarchies shift during stress adaptation requires approaches capable of inferring directional relationships among genes, metabolites, and signaling pathways. Bayesian networks, probabilistic graphical models, and AI-assisted inference methods can integrate multi-omics and environmental datasets to reconstruct regulatory interactions and identify context-dependent changes in network organization (Needham et al., 2007). Unlike static pathway representation, these approaches can infer network rewiring associated with stress-induced changes in signaling and metabolism. This capability is especially relevant because flavonoid biosynthesis is tightly integrated with hormonal signaling, ROS homeostasis, and defense-associated transcriptional cascades. In *Arabidopsis*, interconnected MYB-, CBF, and HOSI-associated regulatory networks coordinate cold-responsive phenylpropanoid activation through transcriptional circuits that simultaneously influence stress signaling and flavonoid biosynthesis (Shkryl et al., 2021). These observations indicate that effective metabolic engineering should target regulatory network organization rather than individual biosynthetic genes alone.

Network analyses must also account for the metabolic constraints that influence flavonoid production. Genome-scale metabolic models and flux balance analysis (FBA) provide a framework for examining how carbon skeleton, reducing power, ATP, and aromatic amino acid precursors are distributed among competing metabolic processes (Sweetlove and Ratcliffe, 2011). Because flavonoid biosynthesis competes with growth, primary metabolism, and other defense pathways for shared resources, enhanced allocation toward anthocyanin or flavonol production may improve stress tolerance while simultaneously affecting carbon utilization and energy balance. Constraint-based modelling therefore provides insight into the trade-offs that shape stress-adaptive metabolic reprogramming and helps identify bottlenecks that may constrain engineering outcomes (Cheung et al., 2014). However, most existing metabolic models have been parameterized using individual stress datasets, limiting their ability to predict resource allocation and metabolic flux under simultaneous biotic and abiotic stress conditions.

Because flavonoid accumulation is often regulated by transient signaling events and feedback mechanisms, static network models provide only a partial view of pathway behavior. Dynamic systems modelling approaches, including ordinary differential equation (ODE)-based models, kinetic modelling, and hybrid AI-driven frameworks, are increasingly being used to predict pathway responses following environmental or genetic perturbation (Kitano, 2002; Chai et al., 2014). By incorporating threshold-dependent activation, feedback regulation, and temporal variation in metabolic states, these models provide a more realistic representation of flavonoid network behavior and generate experimentally testable hypotheses. Their predictive accuracy, however, remains dependent on the availability of high-resolution temporal datasets, which are still scarce for flavonoid responses under combined stress.

An emerging extension of these approaches is the development of biological digital twins for plant metabolic systems (Bai et al., 2024). By integrating genomic, metabolomic, transcriptomic, physiological, phenotypic, and environmental datasets into continuously updated computational frameworks, digital twins may eventually enable simulation of flavonoid pathway behavior across tissues, developmental stages, and stress environments before experimental implementation. Coupling these models with high-throughput phenotyping platforms, environmental sensing systems, and iterative model refinement strategies could substantially improve prediction accuracy under field-relevant conditions (van Dijk et al., 2021). Although still at an early stage in plant biology, these frameworks provide a conceptual foundation for integrating predictive modelling with experimental validation, thereby accelerating the identification of regulatory nodes and metabolic bottlenecks relevant to flavonoid engineering under combined stress.

### 7.4 From network prediction to regulatory target prioritization

The ultimate value of AI-assisted network reconstruction lies in its ability to prioritize regulatory components whose manipulation is most likely to alter flavonoid-associated stress responses. While transcriptomic and multi-omics analyses routinely reveal thousands of stress-responsive genes, only a subset exert sufficient influence over network behavior to modify metabolic outcomes (Kitano, 2002; Tohge et al., 2017). Consequently, target prioritization has become a critical step in translating systems-level knowledge into experimentally tractable engineering strategies.

Target prioritization frameworks increasingly integrate network topology, regulatory connectivity, module membership, and metabolic associations to identify nodes with disproportionate influence over pathway behavior. Unlike conventional approaches that rely primarily on differential expression, these methods evaluate the position of candidate genes within regulatory networks and their contribution to information flow across interconnected pathways (Chezem and Clay, 2016; Chen et al., 2026b). Such analyses frequently prioritize transcriptional regulators over individual biosynthetic enzymes because they coordinate multiple branches of phenylpropanoid metabolism simultaneously. Nevertheless, network centrality alone does not necessarily indicate biological importance. Candidate regulators should therefore be supported by complementary evidence, including functional validation, genetic perturbation, or metabolic phenotyping before they are selected for engineering.

In flavonoid-associated stress networks, MYB, WRKY, NAC, bZIP, and AP2/ERF transcription factors often occupy central positions linking environmental perception with metabolic reprogramming (Chezem and Clay, 2016; Chen et al., 2026b). Because these regulators coordinate transcriptional activity across multiple downstream targets, their perturbation can influence precursor allocation, branch competition, and stress-responsive signaling. For example, manipulation of MYB regulators can alter carbon partitioning between anthocyanin and flavonol biosynthesis, thereby affecting both metabolic composition and adaptive stress responses (Lepiniec et al., 2006; Xu et al., 2015a). Such network-level regulators therefore offer greater potential for coordinated metabolic reconfiguration than modifications confined to individual enzymatic steps.

A further challenge is identifying regulators that retain influence across diverse environmental conditions. Targets identified under single-stress experiments may not govern flavonoid responses under realistic environments where multiple stresses occur simultaneously and extensive interactions arise among hormonal and ROS signaling pathways (Atkinson et al., 2013; Pandey et al., 2017). AI-assisted integration of datasets generated across different stress combinations provides an opportunity to prioritize regulators that consistently emerge despite changes in network state. Such conserved control points may represent more robust engineering targets because they are more likely to contribute to adaptive plasticity than condition-specific responses (Sathishkumar et al., 2026). However, translating computationally prioritized targets into successful engineering strategies remains challenging because regulatory influence predicted in sillico may not always correspond to measurable metabolic or phenotypic outcomes. Taken together, AI-assisted prioritization provides a systematic framework for reducing large and complex biological datasets into a manageable set of high-confidence regulatory candidates. By prioritizing regulators with strong network influence, environmental robustness, and mechanistic relevance to stress adaptation, these approaches establish the rationale for AI-guided CRISPR strategies targeting flavonoid regulatory networks under combined stress.

## 8 AI-driven CRISPR-mediated reprogramming of flavonoid networks under combined stress

### 8.1 AI-enhanced CRISPR platforms for precision engineering of flavonoid networks

CRISPR technologies have transformed genome engineering by enabling precise and programmable manipulation of genetic information across diverse biological systems (Wu et al., 2024; Hina et al., 2025a). Compared with conventional breeding and transgenic approaches, CRISPR-based systems facilitate targeted modification of genomic loci associated with stress adaptation, growth regulation, and specialized metabolic biosynthesis, providing unprecedented opportunities to engineer complex traits with greater precision and efficiency (Jinek et al., 2012; Abbasi et al., 2024). The rapid expansion of the CRISPR toolbox, including Cas9, Cas12, Cas13, base editors, prime editors, and programmable transcriptional regulators, has further broadened the scope of possible genetic interventions, ranging from gene disruption and allele replacement to promoter engineering and multiplex genome editing (Lowder et al., 2015; Nascimento et al., 2023). These advances have established CRISPR as a versatile platform for manipulating complex regulatory and metabolic networks that underpin plant adaptation to environmental stress. This capability enables network-level engineering rather than modification of individual genes.

The potential of CRISPR is particularly relevant for flavonoid biology because flavonoid accumulation is controlled by highly interconnected biosynthetic regulatory, and signaling networks that respond dynamically to environmental cues. Engineering such networks requires precise modification of multiple genetic components, including structural genes, transcription factors, regulatory elements, and signaling regulators (Zhang et al., 2009). However, the increasing complexity of genome-editing objectives has exposed several limitations of conventional CRISPR workflows, including challenges associated with guide RNA selection, unintended off-target activity, and uncertainty in DNA repair outcomes. These constraints become especially significant when editing interconnected metabolic pathways, where unintended modifications may compromise pathway balance, metabolic flux distribution, or stress-responsive regulatory interactions. Consequently, the effectiveness of CRISPR-mediated engineering increasingly depends on predictive frameworks that improve editing precision while reducing experimental uncertainty. This requirement becomes even more important when engineering flavonoid networks under combined stress, where coordinated modification of multiple regulatory components is often required to achieve stable metabolic reprogramming.

AI has emerged as a critical enabling technology for improving the precision and predictability of CRISPR-based genome editing (Barrangou and Doudna, 2016; Chen et al., 2024). Rather than functioning as a standalone editing technology, AI serves as an analytical layer that enhances multiple stages of CRISPR design and implementation. By extracting biologically meaningful patterns from large-scale genomic and genome-editing datasets, machine learning and deep learning algorithms can improve target selection, optimize editing strategies, and predict editing outcomes before experimental validation (**Table 4**). This shift from empirical trial-and-error approaches toward predictive genome engineering is particularly valuable when manipulating complex traits governed by multilayered regulatory networks. By reducing uncertainty before experimental validation, AI also enables more efficient prioritization of editing strategies, thereby lowering the experimental burden associated with multiplex genome engineering.

**Table 4:** Representative AI and ML models for CRISPR design.

Model	Year	AI/ML approach	Primary application	Key features	References
MIT Specificity Score	2013	Rule-based	Off-target prediction	Mismatch-position based specificity estimation	(Hsu et al., 2013)
Wang Score	2014	SVM	On-target prediction	Early ML framework for guide efficacy	(Wang et al., 2014)
CFD Score	2016	Statistical model	Off-target prediction	Mismatch-dependent cleavage probabilities	(Doench et al., 2016)
CRISTA	2017	Random Forest	Off-target prediction	Sequence and genomic context integration	(Abadi et al., 2017)
sgRNA Scorer 2.0	2017	SVM	On-target prediction	Cross-species guide prediction	(Chari et al., 2017)
DeepCRISPR	2018	CNN + Autoencoder	On/off-target prediction	Sequence and epigenetic integration	(Chuai et al., 2018)
DeepCpf1	2018	CNN	On-target prediction	Cas12a guide prediction	(Kim et al., 2018)
DeepCas9	2019	CNN	On-target prediction	Large-scale Cas9 activity learning	(Kim et al., 2019)
CRISPRLearner	2019	Deep CNN	On-target prediction	Automatic feature extraction	(Dimauro et al., 2019)
DeepHF	2019	Deep Neural Network	On-target prediction	High-fidelity Cas9 prediction	(Wang et al., 2019)
CRISPR-Net	2020	CNN + RNN	Off-target prediction	Models mismatches and indels	(Lin et al., 2020)
C-RNNCrispr	2020	CNN + BiGRU	On-target prediction	Local and long-range sequence features	(Zhang et al., 2020b)
Elevation	2020	Ensemble ML	Off-target prediction	Genome-wide risk prediction	(Listgarten et al., 2018)
GuidePro	2021	Ensemble Learning	sgRNA prioritization	Protein knockout optimization	(He et al., 2021)
CRISPRon	2021	Deep Learning	On-target prediction	NGG PAM guide activity	(Xiang et al., 2021)
CRISPRoff	2021	Deep Learning	Off-target prediction	Guide specificity estimation	(Xiang et al., 2021)
AttnToMismatch-CNN	2022	Attention CNN	Off-target prediction	Attention-based mismatch analysis	(Kumar, 2021)
CRISPR-IP	2022	Deep Learning	Off-target prediction	Guide-target pair encoding	(Zhang and Jiang, 2022)
DeepFM-Crispr	2024	DeepFM + DNN	On/off-target prediction	Complex feature interactions	(Bao and Liu, 2024)
Crispr-SGRU	2024	Stacked GRU	Off-target prediction	Mismatch and indel prediction	(Zhang et al., 2024b)
DeepMEns	2025	Deep Ensemble	On-target prediction	Robust sgRNA activity prediction	(Ding et al., 2025)

Among the major challenges in CRISPR applications, off-target activity remains one of the most significant barriers to achieving high editing precision. Although CRISPR-Cas systems are designed to recognize specific DNA sequences, partial sequence similarity elsewhere in the genome can result in unintended cleavage events, potentially generating undesirable genetic modifications (Wang and Zhang, 2019). To address this issue, AI-based prediction models have been developed using large-scale datasets of experimentally validated editing events. Applying ML to CRISPR genome editing requires clearly defining the prediction task. Predicting on-target efficiency constitutes a regression task, while off-target prediction involves classification or ranking (Abadi et al., 2017; Listgarten et al., 2018). These models integrate sequence characteristics and genome-wide editing patterns to identify potential off-target sites with greater accuracy than conventional alignment-based approaches. Deep learning frameworks trained on genome-wide off-target datasets have substantially improved the ability to distinguish highly specific target sites from those prone to unintended editing (Lin and Wong, 2018; Listgarten et al., 2018). Tools such as DeepCRISPR and CRISPR-Net exemplify this transition toward predictive editing design by enabling the identification and prioritization of target sites with reduced off-target risk before experimental implementation (Chuai et al., 2018; Lin et al., 2020). The performance of DeepCRISPR illustrate how AI-assisted guide selection can improve genome editing while reducing the likelihood of unintended editing events (Kleinstiver et al., 2016).

AI is also reshaping guide RNA (gRNA) design, a critical determinant of CRISPR efficiency and accuracy (Liu et al., 2020). The effectiveness of genome editing depends heavily on the ability of guide RNAs to direct nuclease activity toward the intended genomic target while minimizing unintended interactions. Because guide performance is influenced by multiple sequence and contextual features, identifying optimal guides through empirical screening alone can be laborious and inefficient. Machine learning algorithms trained on extensive editing datasets can identify sequence patterns associated with highly effective guides, enabling prediction of editing performance prior to experimentation (Chuai et al., 2018; Zhang et al., 2020a). Consequently, AI-assisted platforms such as CRISPR AI and sgRNA Designer facilitate the selection of guide RNAs with improved efficiency and specificity, thereby increasing editing success while reducing experimental costs and screening requirements (Dai et al., 2024). Emerging domain-specific platforms further extend these capabilities to specialized metabolic pathways. For example, FlavoCRISPR-AI combines genomic mining, deep-learning-assisted guide RNA prediction, target-specificity assessment, and in silico biochemical analyses to support editing of flavonoid-associated genes (Bhadana et al., 2026). By combining target discovery with CRISPR design optimization, such platforms provide a useful framework for integrating regulatory target prioritization with precision genome editing.

The integration of AI has also accelerated the development and deployment of next-generation editing systems, including base editors. Unlike conventional CRISPR-Cas9 approaches that rely on double-strand DNA cleavage, base editors enable direct nucleotide substitutions without generating double-strand breaks, thereby reducing the risks associated with error-prone DNA repair (Kantor et al., 2020). Nevertheless, editing efficiency and specificity remain strongly influenced by local sequence context and editing-window characteristics. AI-driven predictive models have therefore been developed to estimate editing probabilities, identify optimal target positions, and evaluate potential off-target modifications, enhancing the reliability of base-editing applications (Dimauro et al., 2019; Marquart et al., 2021; Xiang et al., 2021; Dixit et al., 2024). Such capabilities are particularly valuable when engineering subtle regulatory variants or functional alleles that influence metabolic pathways and stress-responsive processes.

Beyond target recognition and editing efficiency, AI is increasingly being used to predict the molecular consequences of genome editing (Liu et al., 2020). A major source of uncertainty in CRISPR applications arises from cellular DNA repair mechanisms, which can generate diverse insertion-deletion (indel) outcomes following nuclease-induced cleavage. Predicting these repair outcomes is essential for achieving desired genetic modifications and minimizing unintended consequences. Machine learning-based platforms such as InDelphi and FORECasT utilize large experimental datasets to model DNA repair behavior and forecast the spectrum of editing outcomes likely to arise from a given intervention (Shen et al., 2018; Allen et al., 2019). Another deep learning model, Apindel, predicts the majority of Cas9-generated mutational outcomes and reveals how nucleotide composition surrounding the cleavage sites influences CRISPR/Cas9 editing outcomes (Liu et al., 2022b). Together, these predictive models enable estimation of DNA repair outcomes before experimental implementation, thereby improving the efficiency and reliability of genome-engineering strategies. Collectively, these developments position AI as an enabling layer that improves the precision, predictability, and scalability of CRISPR-based genome engineering. However, most AI-assisted CRISPR platforms have been developed and validated using general genome-editing datasets rather than flavonoid-specific regulatory networks or combined stress systems, expanding these frameworks to incorporate stress-context-dependent regulatory information will therefore be essential for realizing their full potential in flavonoid metabolic engineering. Within this context, AI-assisted prioritization of regulatory nodes, combined with increasingly precise genome-editing platforms, provides a rational framework for reconfiguring flavonoid regulatory networks to enhance adaptation under combined biotic and abiotic stress.

### 8.2 AI-CRISPR-mediated reprogramming of flavonoid metabolic flux

Flavonoid biosynthesis is organized as a highly branched metabolic network in which multiple end products compete for common precursors (Saito et al., 2013). Consequently, engineering outcomes are frequently determined by the redistribution of carbon among competing pathway branches rather than by changes in overall pathway activity. AI-assisted analyses of integrated genotype-phenotype-metabolome datasets help identify regulatory and enzymatic nodes with disproportionate influence over pathway behavior, thereby improving genome-editing target selection (Wada et al., 2020). Under combined biotic and abiotic stress, metabolic demands continuously shift as plants balance antioxidant defence, antimicrobial responses, and growth. AI-guided prioritization of flux-control nodes therefore provides a strategy to redirect carbon allocation towards flavonoid subclasses that best support these competing physiological demands, rather than simply increasing total flavonoid accumulation.

Among the most influential control points are branch-point enzymes that compete for shared intermediates and directly determine carbon allocation within the pathway. A well-characterized example is the competition between DFR and FLS for dihydroflavonol substrates. The relative activities of these enzymes determine whether metabolic flux is directed toward anthocyanin or flavonol biosynthesis, making this node a major regulator of flavonoid composition (Luo et al., 2016; Choudhary and Pucker, 2024). Biochemical analysis in *Rubus chingii* demonstrated that FLS exhibits greater affinity for dihydroflavonol substrates than DFR and that flavonol can further inhibit DFR activity, reinforcing the competitive relationship between these enzymes (Lei et al., 2023). Comparative studies across rose, petunia, carnation, azalea, and camellia have similarly associated elevated FLS expression with white-flowered phenotypes, whereas increased DFR activity is typically linked to anthocyanin accumulation and red pigmentation (Luo et al., 2016). Although these studies were conducted primarily in pigmentation systems, they establish mechanistic principles for manipulating carbon portioning between competing flavonoid branches. Such branch points are particularly relevant under combined stress, where adaptation may depend on selective enrichment of specific flavonoid subclasses rather than uniform activation of the entire pathway. These findings suggest that branch-point enzymes function as metabolic decision points, making them attractive targets for AI-guided CRISPR strategies aimed at reshaping flavonoid composition according to stress-specific demands.

Experimental studies provide direct evidence that modification of branch-point enzymes can redirect flavonoid metabolism. In petunia, constitutive expression of DFR combined with antisense suppression of FLS substantially increased anthocyanin accumulation, demonstrating that coordinated manipulation of competing enzymes can alter pathway output in a predictable manner (Davies et al., 2003). Similar outcomes have been achieved through genome editing. For example, Watanabe et al. (2017) used CRISPR/Cas9 to disrupt the *DFR-B* locus in *Ipomoea nil*, resulting in white-flowered phenotypes in 75% of transgenic plants carrying biallelic mutations. These studies demonstrate that branch-point enzymes function as effective metabolic control nodes whose manipulation can predictably alter carbon allocation within flavonoid biosynthesis. These experimentally validated control points support AI-guided prioritization of metabolic intervention points with the greatest influence on pathway behavior, providing a rational basis for CRISPR-mediated flux engineering.

Flux reprogramming is not restricted to enzymes operating within the flavonoid pathway itself. Because flavonoid biosynthesis is embedded within the broader phenylpropanoid network, alterations in competing pathways can substantially influence flavonoid accumulation. Manipulation of upstream branch-point enzymes such as cinnamoyl-CoA reductase (CCR), which catalyzes a key step in lignin biosynthesis, can reduce carbon allocation to lignin and increase the availability of phenylpropanoid intermediates for alternative secondary metabolic pathways, including flavonoid biosynthesis. These observations demonstrate that pathway flux can also be reconfigured through interventions at upstream control points within the phenylpropanoid pathway (Vanholme et al., 2010; Barakat et al., 2011). Likewise, transgenic tobacco overexpressing tea-derived DFR or ANR accumulated higher flavonoid levels and exhibited enhanced antioxidant capacity, illustrating how modification of downstream enzymatic steps can alter flux distribution within flavonoid biosynthesis and influence plant physiological traits (Kumar et al., 2013). These findings highlight that engineering stress-responsive flavonoid profiles may require coordinated modification of both flavonoid-specific enzymes and upstream carbon-allocation pathways to maintain metabolic balance under combined stress.

These observations are consistent with constraint-based modeling and flux-balance analyses, which indicate that metabolic phenotypes frequently emerge from network-wide redistribution of flux rather than from the activity of individual genes in isolation (Sweetlove and Ratcliffe, 2011; Yuan et al., 2016). White-testa peanuts provide a compelling example of this principle, exhibiting a substantial reduction in anthocyanins and proanthocyanidins (PAs) together with increased accumulation of flavonols, flavone C-glycosides, and upstream flavonoid intermediates, suggesting a redistribution of metabolic flux within the flavonoid network (Wan et al., 2020; Huai et al., 2025; Chen et al., 2026c). Such coordinated metabolic shifts underscore the importance of identifying pathway nodes that exert disproportionate control over carbon allocation. Recent advances in metabolic network modeling further demonstrate that integrating genome-scale metabolic models with multi-omics datasets can predict pathway bottlenecks, quantify flux redistribution, and prioritize candidate intervention points within complex plant metabolic networks. Under combined stress conditions, where plants continuously redistribute metabolic resources in response to interacting environmental signals, AI-guided identification of flux control nodes provides a rational framework for CRISPR-mediated reprogramming of flavonoid metabolism. Rather than maximizing flavonoid accumulation, this strategy seeks to optimize the composition and distribution of flavonoid subclasses to enhance stress resilience while maintaining overall metabolic balance.

### 8.3 AI-guided multiplex editing for network-level reconfiguration

A major challenge for flavonoid engineering is that adaptive responses often depend on coordinated regulation of multiple pathways rather than modification of individual genes. Consequently, editing strategies based on single regulators frequently fail to reproduce coordinated metabolic responses. AI-assisted network reconstruction frequently identifies groups of interacting regulators rather than single dominant control points. Graph-learning approaches can therefore prioritize combinations of transcription factors, metabolic enzymes, and transport components that collectively determine flavonoid responses, thereby providing a rationale for multiplex genome editing (Caradus, 2023). This represents a shift from editing individual regulators to reconfiguring coordinated regulatory modules that underpin flavonoid adaptation under combined stress.

Another challenge concerns the prevalence of epistatic interactions within stress-responsive regulatory networks. The phenotypic outcome of simultaneously modifying multiple loci is frequently non-additive because regulatory pathways converge on shared signaling and metabolic modules (Phillips, 2008). Large scale genetic interaction studies have demonstrated that synergistic and antagonistic relationships among genes can dramatically alter phenotypic outcomes and limit the predictive power of single-gene analyses (Costanzo et al., 2016). This issue is particularly relevant for flavonoid engineering because regulators controlling secondary metabolism often influence growth, development, and defense simultaneously (Agati and Tattini, 2010; Xu et al., 2015a). Therefore, editing combinations that appear beneficial when evaluated individually may produce unexpected trade-offs when combined. Machine-learning models trained on multi-omics and genome editing datasets provide a framework for predicting higher-order interactions and reducing the combinatorial search space associated with multiplex genome engineering (Caradus, 2023). AI-guided prediction of higher-order interactions therefore provides a practical strategy for selecting multiplex editing combinations with greater likelihood of producing desirable metabolic outcomes.

The emergence of multiplex CRISPR technologies provides the experimental capacity to implement such network-level interventions. Contemporary systems permit simultaneous modifications of multiplex genomic loci through combinations of gene knockout, promoter editing, CRISPR activation, and CRISPR interference strategies (Lowder et al., 2015; Zhang et al., 2016; Zetsche et al., 2017). Within flavonoid pathways, this capability creates opportunities. For example, AI-guided editing strategies could combine promoter editing of stress responsive MYB regulators, fine-tuning of FLS-DFR competitive flux points, and manipulation of vacuolar transport systems within a single multiplex construct. Such interventions move beyond conventional pathway engineering towards coordinated reprogramming of stress-adaptive network states. AI-assisted approaches therefore provide a framework for coordinating genome-editing interventions across multiple regulatory layers that contribute flavonoid biosynthesis, signaling, transport, and deployment under stress conditions. As our understanding of flavonoid regulatory networks improves, such strategies may facilitate the development of crops with more coordinated flavonoid responses under combined stresses. These advances establish the foundation for iterative optimization frameworks in which genome-editing outcomes are systematically evaluated and incorporated into subsequent design cycles.

### 8.4 Design–build–test–learn cycles for predictive flavonoid engineering

As discussed above, AI-assisted target prioritization and CRISPR-based perturbation strategies provide a framework for reprogramming flavonoid regulatory networks. However, the predictive accuracy of these approaches ultimately depends on how effectively experimental outcomes are incorporated into subsequent rounds of model refinement. Therefore, predictive engineering requires not only accurate target selection but also systematic mechanisms for updating biological models as new experimental evidence becomes available (Radivojević et al., 2020).

In synthetic biology, Design–Build–Test–Learn (DBTL) frameworks provide an iterative strategy for engineering complex biological systems through continuous integration of computational prediction and experimental validation (Carbonell et al., 2019). The workflow typically begins with a design phase in which biological hypotheses are formulated and computational tools identify candidate interventions. This is followed by construction of engineered genotypes, experimental evaluation of system performance, and incorporation of the resulting data into subsequent rounds of model refinement. Recent advances in artificial intelligence have substantially enhanced the design stage by enabling extraction of biologically meaningful patterns from large-scale datasets, prioritization of candidate targets, and prediction of engineering outcomes before experimental implementation (Marucci et al., 2020; Lawson et al., 2021).

**Figure 5:**
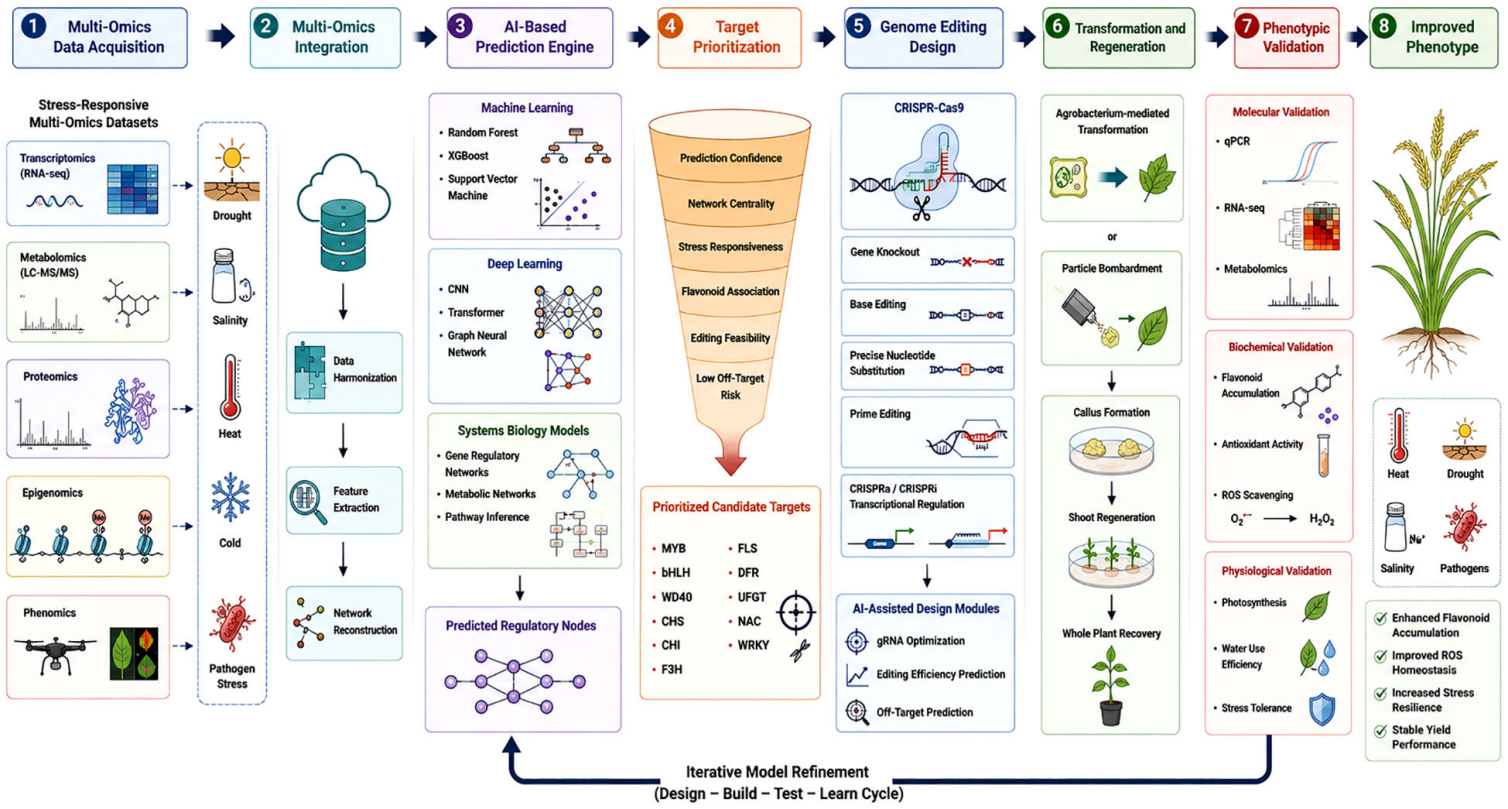
Integrated AI-guided framework for translating stress-responsive multi-omics information into precision genome editing strategies aimed at enhancing flavonoid-mediated stress adaptation in plants. The workflow begins with the acquisition of transcriptomic, metabolomic, proteomic, epigenomic, and phenomic datasets generated under diverse abiotic and biotic stress conditions. Following multi-omics integration and network reconstruction, AI-based analytical approaches identify regulatory genes and pathways associated with flavonoid biosynthesis and stress adaptation. Candidate targets are subsequently prioritized using biological relevance and editability criteria before being subjected to CRISPR-based genome engineering. Regenerated edited plants undergo comprehensive molecular, biochemical, and physiological validation to confirm improved flavonoid accumulation, antioxidant capacity, and stress tolerance. The incorporation of a Design–Build–Test–Learn (DBTL) feedback loop enables continuous refinement of predictive models, thereby accelerating the development of climate-resilient crop varieties with enhanced adaptive potential.

The relevance of DBTL frameworks to flavonoid engineering arises from the inherent complexity of flavonoid regulation under combined stress. AI models can identify candidate intervention points from integrated omics datasets. However, the biological consequences of these interventions often become apparent only after experimental implementation and stress evaluation (Zandalinas et al., 2021), highlighting the need for iterative model refinement. Consequently, the outcomes of CRISPR-mediated modifications provide and important source of information for improving subsequent prediction cycles (**Figure 5**). Within DBTL frameworks, AI-prioritized targets identified from integrated transcriptomic, metabolomic, and regulatory network analyses can be implemented through genome-editing strategies such as promoter engineering, multiplex editing, or transcriptional reprogramming. Following the build phase, engineered lines can be evaluated under relevant combined-stress conditions using high-throughput phenotyping together with metabolomics, and transcriptomic profiling. Such approaches increasingly recognized as essential for capturing the systems-level consequences of metabolic engineering interventions (Furbank and Tester, 2011; Tardieu et al., 2017). Measurements of flavonoid composition, pathway activity, and stress adaptation can then be compared with model predictions to identify discrepancies between expected and observed responses. Such deviations often reveal previously unrecognized regulatory bottlenecks, metabolic constraints, or context-dependent interactions that are difficult to infer from pre-editing datasets alone. For example, edits predicted to enhance flux toward a particular flavonoid subclass may produce weaker-than-expected responses because precursor availability, transport capacity, or competing phenylpropanoid branches impose additional constraints on pathway behavior. By incorporating experimentally validated outcomes into subsequent rounds of model training, DBTL frameworks enable continuous refinement of network architecture and target prioritization. Similar iterative learning strategies have improved predictive accuracy and design efficiency in synthetic biology and metabolic engineering systems (Carbonell et al., 2019; Radivojević et al., 2020). Applied to flavonoid engineering, iterative learning cycles could improve the prediction of multiplex editing outcomes, facilitate identification of robust regulatory targets, and support increasingly precise reprogramming of flavonoid metabolism under combined biotic and abiotic stress conditions.

## 9 Current research gaps and translational potential

The preceding sections highlight that flavonoid-mediated stress adaptation cannot be explained by individual biosynthetic reactions or isolated signaling pathways. Instead, it emerges from the coordinated interaction of metabolic branch competition, spatiotemporal regulation, hormone crosstalk, and multilayered transcriptional control. Although these mechanisms have been investigated extensively, an integrated understanding of how they collectively regulate flavonoid responses under combined biotic and abiotic stress is still lacking. As a result, it remains difficult to determine which regulatory processes consistently contribute to successful adaptation under combined stress conditions.

Most mechanistic studies have focused on individual regulatory processes, whereas comparatively few have examined how metabolic flux, signaling pathways, and transcriptional regulation are dynamically coordinated under combined stress (Atkinson et al., 2013; Zandalinas et al., 2021). Consequently, the relative contribution of different flavonoid subclasses, the stability of regulatory networks, and the mechanisms that prioritize metabolic allocation under changing environmental conditions remain poorly resolved (Agati et al., 2020). Addressing these questions will be essential for distinguishing core adaptive mechanisms from responses that are specific to individual experimental conditions.

Another important limitation is the persistent gap between statistical association and biological causality. High-throughput transcriptomic, metabolomic, and network analyses have identified numerous genes, metabolites, and regulatory modules associated with flavonoid biosynthesis and stress responses (Chen et al., 2020; Song et al., 2022). However, many proposed regulators remain supported primarily by co-expression patterns or network connectivity rather than direct functional validation (Usadel et al., 2009). This limitation becomes even more pronounced under combined stress, where multiple signaling pathways operate simultaneously and regulatory functions are often context dependent. Consequently, relatively few candidate regulators have been validated across different stress combinations, crop species, and environmental conditions. Greater integration of reverse genetics with quantitative physiological analyses and rigorous functional validation will therefore be essential for distinguishing causal regulators from downstream stress-responsive markers.

The growing availability of multi-omics datasets has transformed flavonoid research, but data integration remains a significant challenge. Transcriptomic, proteomic, metabolomic, and epigenomic datasets are frequently generated using different experimental designs, developmental stages, and environmental conditions, limiting their direct comparability (Subramanian et al., 2020; Roychowdhury et al., 2023). This lack of standardization reduces the ability to reconstruct dynamic regulatory networks and restricts the development of predictive models that accurately reflect biological behavior. Future efforts should therefore emphasize standardized experimental frameworks, interoperable databases, and integrated analyses that connect molecular regulation with physiological performance under combined stress conditions.

These limitations directly influence the application of artificial intelligence and genome editing. However, the reliability of these predictions depends on the quality and biological diversity of the underlying data (Mostafa et al., 2023). The predictive performance of current models is constrained by the limited availability of comprehensive, high-quality multi-omics datasets generated under multifactorial stress environments (Libbrecht and Noble, 2015). This limitation restricts their ability to accurately capture the dynamic regulatory interactions governing flavonoid-mediated stress responses in complex environments. Similarly, although CRISPR technologies provide powerful tools for manipulating flavonoid-associated genes, the phenotypic consequences of individual edits are frequently varied due to the context-dependent nature of gene function and metabolic regulation (Voytas and Gao, 2014). Improving the predictive value of AI and the stability of genome-editing outcomes will therefore require validation across diverse biological and ecological context (Han and Liu, 2022).

Beyond scientific challenges, successful deployment of flavonoid-engineered crops will depend on overcoming regulatory, ecological, and socioeconomic barriers. Although several countries have adopted more permissive regulatory frameworks for genome-edited crops, international policies remain inconsistent, creating uncertainty for breeding programs and technology transfer (Hundleby and Harwood, 2022; Idris et al., 2023). Comprehensive assessment of unintended metabolic changes, ecological interactions, and long-term environmental consequences will remain essential for responsible implementation (Ayanoğlu et al., 2020). Public acceptance similarly depends on transparent communication of benefits, potential risks, and equitable access to emerging technologies rather than scientific innovation alone (Lassoued et al., 2019). Furthermore, expanding intellectual property surrounding CRISPR platforms, AI-assisted breeding tools, and associated datasets may disproportionately restrict their adoption within public breeding programs and low-resource agricultural systems (Pfeiffer et al., 2018). Addressing these governance and accessibility issues will therefore be integral to ensuring that technological advances translate into broadly applicable agricultural solutions.

Collectively, these knowledge gaps demonstrate that the principal challenge is no longer the discovery of additional flavonoid-associated genes or metabolites, but identifying which regulatory mechanisms consistently govern flavonoid-mediated adaptation under combined stress conditions. Resolving this challenge will require causal validation of candidate regulators, standardized multi-omics resources, biologically informed AI models, and rigorous evaluation across diverse genetic backgrounds and field environments. Addressing these priorities will accelerate the translation of mechanistic insights into robust strategies for developing crops with durable resilience to increasingly complex environmental stresses.

## 10 Conclusion

Overall, this review highlights that flavonoids contribute to plant adaptation through diverse biochemical, physiological, and regulatory mechanisms. Their structural diversity and dynamic regulation enable them to perform multiple biological functions. They act as natural deterrents against herbivores and pathogens while contributing to redox homeostasis, membrane stability, osmotic regulation, and stress-responsive signaling. These complementary roles demonstrate that flavonoid-mediated defense extends well beyond antioxidant activity and emerges from the coordinated physiological and metabolic regulation.

A central theme emerging from this review is that flavonoid responses cannot be fully understood by examining individual stresses in isolation. Under combined biotic and abiotic stress, flavonoid metabolism is reprogrammed through dynamic interactions among metabolic regulation, hormone signaling and environmental cues. As a result, flavonoid accumulation is shaped not only by stress intensity but also by stress combinations, developmental stage, tissue specificity, and the timing of stress exposure. These context-dependent responses highlight the importance of investigating flavonoid regulation under multifactorial conditions that more closely resemble agricultural environments.

An important conclusion emerging from this review is that multi-omics, artificial intelligence, genome-editing technologies, and DBTL frameworks should not be viewed as independent tools but as complementary components of an integrated research platform for predictive flavonoid engineering. Together, they provide new opportunities to resolve the regulatory complexity of flavonoid networks, prioritize biologically meaningful engineering targets, and accelerate the development of crops with improved resilience to combined biotic and abiotic stresses. Their greatest value lies not in generating more data, but in translating mechanistic understanding into predictive and experimentally testable strategies for crop improvement.

Despite these advances, important barriers continue to limit practical application. Much of the current evidence is derived from controlled experiments, with relatively few studies evaluating flavonoid regulation under realistic combinations of biotic and abiotic stress. The stability of engineered traits across diverse biological and environmental contexts remains uncertain. In addition, the long-term metabolic and ecological consequences of modifying flavonoid pathways are still poorly understood. Bridging these gaps will require rigorous functional validation, multi-environment field evaluation, and iterative testing of computational predictions with experimentation.

Overall, this review highlights the need to move beyond descriptive studies of flavonoid accumulation towards a systems-level understanding of flavonoid regulation under combined stress. Rather than focusing solely on increasing flavonoid accumulation, future engineering strategies will likely depend on the ability to coordinate regulatory networks, metabolic fluxes, and stress-responsive signaling processes in a context-dependent manner. By integrating advances in stress biology, multi-omics analyses, artificial intelligence, genome editing, and DBTL frameworks, the approaches discussed here provide a foundation for more informed and biologically grounded engineering strategies. Future research should focus on identifying regulatory mechanisms that remain effective across diverse stress combinations and agricultural settings while strengthening the integration of computational prediction with experimental validation. Progress in these areas will support the development of resilient crop varieties and strengthen agricultural sustainability in the face of environmental uncertainty.
